# The dimensionality of the Conflict Resolution Styles Inventory across age and relationships

**DOI:** 10.3389/fpsyg.2024.1233279

**Published:** 2024-03-12

**Authors:** Tatiana Alina Trifan, Wim Meeus, Susan Branje

**Affiliations:** ^1^The Department of Youth and Family, Utrecht University, Utrecht, Netherlands; ^2^School of Behavioural, Social and Legal Sciences, Örebro University, Örebro, Sweden

**Keywords:** conflict management, relationships, adolescents, bifactor, adults

## Abstract

Close interpersonal conflicts between parents and children, marital or romantic partners, and between friends are common, and adjustment in youth and adults depends on how these conflicts are managed. While conflict management is important for relationships and adjustment, the structure of conflict management in adults or in youths has rarely been examined. Knowing how conflict management is structured, and whether this structure changes with age and relationships, is important to understanding what factors influence the development of conflict management skills, and how to intervene. In the current study, we explored the unidimensional vs. multidimensional structure of conflict management in family relationships, friendships and romantic relationships across adolescence and adulthood. The sample consisted of 497 Dutch adolescents (57% boys, *M*_age_ = 13.03, SD = 0.46, 11–15 years old) who were followed over 11 years in 9 measurement waves, and their parents, siblings, best friends (six waves), and romantic partner (three waves). First-order factor analyses (CFA) showed that the structure of conflict management is similar for adolescents and adults, across relationships. The results of second-order models, including the theoretical higher dimensions positive/negative conflict management and engagement/disengagement, showed no support for these higher dimensions. The results of bifactor models showed differences between adults and youths: while positive problem solving was part of the general factor of conflict management in adults, it was not part of this general factor in adolescents. The general factor was linked to increases in internalizing and externalizing problems, and with decreases in prosocial behavior. Overall, the bifactor models increased the interpretability and validity of the conflict management measure.

## 1 Introduction

Interpersonal conflict in close relationships is common. Such relationships are those with parents and peers in adolescence (Adams and Laursen, 2001), as well as those between romantic partners and with children (Laursen et al., 1998). The adjustment of both youth and adults depends on the way these conflicts are handled. Negative conflict management with family members, close friends and romantic partners has been linked to youths' externalizing and internalizing problems (e.g., delinquency, van Doorn et al., 2008; Branje et al., 2009; couple violence, Bonache et al., 2016; victimization, Bonache et al., 2016; depression, Boersma-van Dam et al., 2017). Positive conflict management strategies were linked to better adjustment (e.g., longer relationships, Shulman et al., 2008; empathy, Van Lissa et al., 2016) and fewer conflicts (e.g., Missotten et al., 2017). The way interpersonal conflicts are handled is, thus, very important.

Nevertheless, studies differ in the conflict management dimensions they distinguish. Whereas, some studies measured conflict management as being unidimensional, focusing on only positive or negative conflict solving (e.g., D'Zurilla et al., 2004; Castellani et al., 2014), others explored a range of conflict management dimensions (e.g., Kurdek, 1994; Shulman et al., 2008). While the theory on conflict management agrees on the existence of axes such as positive vs. negative, and engagement vs. disengagement (e.g., Laursen, 1993), there is no unified view of the basic structure of conflict management. Studies developed measures of conflict management either to test other hypotheses (e.g., problem solving, D'Zurilla et al., 2004) or to focus only on romantic relationships (e.g., Kurdek, 1994; Bonache et al., 2016; Fortin et al., 2020), rather than to test the structure of conflict management itself more broadly. Given that conflict management dimensions can be simultaneously characterized as positive vs. negative and as engaging vs. disengaging (Laursen, 1993), an assessment of the structure of conflict management needs to include more dimensions. Also, a broader range of age groups and types of relationship would offer a more complete image of the structure of conflict management. Knowing how conflict management is structured, and whether this structure is different in adolescents and adults and in vertical and horizontal relationships, is important to understanding how conflict management affects adjustment, what factors influence the development of conflict management skills, and how to intervene. In the current study, we explored the structure of conflict management in parent-adolescent relationships, from parent and adolescent perspectives, in friendships, and partner relationships of adolescents and adults. We compared the structure of conflict management via first-order, second-order, and bifactor measurement models.

Interpersonal conflict management is defined as the way a disagreement between two persons is handled (Shantz, 1987). From a theoretical perspective, interpersonal conflict management strategies can be characterized on two axes: positivity and engagement (Laursen, 1993). Based on the combinations between these two theoretical axes, four conflict management dimensions stand out across the literature: *positive problem solving*, a positive conflict management dimension based on engagement which includes strategies such as compromise and negotiation (e.g., compromise, Rubenstein and Feldman, 1993; negotiation, Kurdek, 1994; Laursen et al., 2001; nonaggression, Unger et al., 2003; Rodríguez-Ruiz et al., 2015; conciliatory remarks, Ferrar et al., 2020); *conflict engagement*, a negative conflict management dimension that includes coercion tactics such as personal attacks, verbal abuse, and anger (e.g., attack, Rubenstein and Feldman, 1993; conflict engagement, Kurdek, 1994; coercion, Laursen et al., 2001; physical and non-physical aggression, Unger et al., 2003; dominance, Rodríguez-Ruiz et al., 2015; disagreement and confrontative remarks, Ferrar et al., 2020); *withdrawal*, a negative conflict management dimension that involves disengaging from conflict strategies such as tuning the other person out, avoidance, refusing to discuss (e.g., avoidance, Rubenstein and Feldman, 1993; Kurdek, 1994; disengagement, Laursen et al., 2001; Rodríguez-Ruiz et al., 2015; withdrawal, Ferrar et al., 2020); and *compliance*, which involves disengagement from conflict via giving in without defending one's position (see Kurdek, 1994; Branje, 2008;). Based on the theory of conflict management that posits two axes of conflict management strategies, that is, positive vs. negative and engaging vs. disengaging (Laursen, 1993), we chose a measure that has basic dimensions that reflect this theory. This measure is the Conflict Management Style Inventory (CRSI, Kurdek, 1994).

These four conflict management dimensions are useful indicators of youths' and adults' adjustment across age and type of relationship. Higher levels of conflict engagement across relationships negatively impacted youths' social development from childhood to early adulthood (Laursen, 1993; Kim et al., 2001). Conflict engagement and withdrawal within the family were linked to a coercive family environment, to higher levels of conflict between parents and children (e.g., Missotten et al., 2017) and to lower relationship satisfaction (van Doorn et al., 2008). In romantic couples, high levels of conflict engagement were linked to anxiety and partner abuse (see Bonache et al., 2016, 2017), and with couple dissolution (e.g., Gottman, 1993). Positive problem solving, on the other hand, was linked to supportive parenting (e.g., Missotten et al., 2018), maternal responsiveness and youths' agreeableness (Missotten et al., 2017). Compliance was linked to internalizing problems (e.g., Branje et al., 2009; Hanzal and Segrin, 2009) and higher arousal (Perrone-McGovern et al., 2013), but was also found to reduce the influence of avoidant attachment on relationship quality (e.g., Sierau and Herzberg, 2012). Thus, while negative conflict management is linked to hostile environments and adjustment problems, positive conflict management is linked to supportive environments and positive development.

### 1.1 The construct of interpersonal conflict management

From a theoretical perspective, interpersonal conflict management dimensions can be differentiated on two theoretical axes: positivity and engagement (Laursen, 1993). The separate dimensions such as negotiation, coercion and withdrawal, while distinct, have commonalities on the positive – negative axis (Laursen, 1993). Dimensions such as negotiation are theoretically considered as positive conflict management, while conflict engagement and withdrawal are considered as negative conflict management (Rubenstein and Feldman, 1993; Kurdek, 1994, 1995). This suggests shared commonalities, and that the structure of conflict management could be hierarchically reduced to two dimensions: positive vs. negative conflict management (Fortin et al., 2020). Conflict management can also be classified on the axis of engagement vs. disengagement (see Laursen, 1993). Negotiation and conflict engagement involve engagement in handling the conflict, while compliance and withdrawal involve disengagement from handling the conflict (Kurdek, 1994, 1995). This delineation suggests that the components of conflict management construct share commonalities, thus implying a higher-order structure of the construct, with engagement as second-order factor. So far, except for one study exploring only the positive vs. negative axis (Fortin et al., 2020), these theoretical distinctions in the conflict management strategies based on the higher dimensions positivity and engagement have not been tested empirically. In the current study, as a first step in the hierarchical exploration of the construct, we will explore this second-order structure of conflict management to see if the higher-order theoretical dimensions are confirmed.

While theory distinguishes between higher dimensions such as positivity and engagement, at empirical level, the two higher-order dimensions of positivity and engagement might not be enough to explain the structure of conflict management. The pattern of correlations between conflict management dimensions reported across studies indicates the existence of common variance across all dimensions, as well as unique contributions of each dimension (Kurdek, 1995; Missotten et al., 2017). Moreover, theory suggests the dimensions do not completely overlap (Laursen et al., 2001), which means that we cannot disregard the unique contributions of each dimension (Rodriguez et al., 2016). A second-order dimension is based on the correlations between the first-order dimensions, and focuses on what the dimensions have in common (Chen et al., 2006). This means that a second-order dimension says little about the specific contribution of each dimension. A bifactor model is more useful to understand the common variance between the dimensions, while accounting for their specificity. A bifactor model posits both the existence of a general dimension, which accounts for the common variance between the observed behaviors (items), and the existence of specific dimensions which account for the variance in items that is not accounted by the general dimensions (Chen et al., 2006). As all dimensions of conflict management correlate with each other (e.g., Kurdek, 1995; Missotten et al., 2017), this hints to common variance, thus an underlying general factor (Rodriguez et al., 2016). At the same time, the correlations between dimensions are mostly average, which suggests that there is a unique influence of each specific dimension, aside from their commonalities accounted by the general factor of conflict management (Chen et al., 2006). Overall, we hypothesize a bifactor structure might be more adequate for understanding the structure of conflict management than a second-order model.

The construct of conflict management has non-interchangeable dimensions that have commonalities on the axes engagement-disengagement and positive-negative (see Kurdek, 1995). A traditional bifactor model assumes that dimensions are interchangeable, and as such is not the most appropriate model for testing the commonalities between non-interchangeable dimensions. A modified bifactor model that takes into account the non-interchangeable nature of the dimensions of conflict management, such as a bifactor model with reference domain, is more adequate (see Eid et al., 2017, 2018). Thus, we chose such a model for our study. The choice of reference domain depends on the theory as well as the scope of the research (see Eid et al., 2018). For conflict management, as positive strategies are less likely to be followed by negative strategies, and vice-versa during the same conflict (see Laursen, 1993), and as people are more likely to alternate between engagement and disengagement (see Laursen, 1993), as reflected also by the correlations of other dimensions with withdrawal (Kurdek, 1995; Missotten et al., 2017), we chose withdrawal as reference domain and meaning for the general factor. In sum, we used a bifactor model with withdrawal as reference domain when exploring whether the commonalities between the dimensions can be explained by a general factor.

Thus, we explored whether the construct of conflict management has a hierarchical structure, in which the separate dimensions are subordinated to the higher dimensions positivity and engagement, or whether there is a general factor of conflict management with a meaning given by withdrawal as reference domain, which accounts for the commonalities between the four dimensions found by previous studies, and three specific factors.

### 1.2 Conflict management strategies from childhood to adulthood

Which conflict management strategies are used changes over time, from childhood to adulthood, paralleling youths' cognitive and emotional development, increases in perspective taking, and diversification of social environments from childhood until early adulthood (e.g., Smetana et al., 1991). Compromise and negotiation require abstract cognitive skills such as empathy and perspective taking, and surface later during development, replacing the coercive strategies (e.g., Laursen et al., 2001). In relationships with peers, the use of coercive conflict management decreases with age (Laursen et al., 2001), making place for negotiation and disengagement in late adolescence (Laursen et al., 2001). The same pattern has been found in parent-child relationships. The use of coercive conflict management in the parent-child dyad decreased over time as reported by both youths and parents, while negotiation and withdrawal increased from early to late adolescence (Laursen et al., 1998; van Doorn et al., 2011b). Overall, cognitive developments from childhood to early adulthood facilitate the increase in the use of less coercive conflict management skills in relationships both within and outside the family.

While studies have addressed the changes in the use of different dimensions of conflict management over time (e.g., van Doorn et al., 2011b), very few studies explored whether the structure of the construct itself differs between age groups. Studies on the structure of conflict management did not explore if the content or number of dimensions differs across age groups. Some of these studies noted some difficulties to replicate a set number of dimensions in different age groups in cross-sectional data (see adolescents, Bonache et al., 2016; adults, Kurdek, 1995). This suggests that there might be differences in the structure between adolescents and adults. Given the developmental changes throughout adolescence and young adulthood (e.g., Smetana et al., 1991), it is not unlikely that conflict management strategies in adolescents, youths and adults might differ not only in prevalence, but also in structure.

In the current study we examined whether there are four distinct conflict management dimensions from early adolescence to adulthood, or whether some dimensions start as undifferentiated from each other, only to become distinct later in life. The fact that youths' use of conflict management changes over time, that is, older adolescents shift from handling conflict via aggressive behaviors to handling conflict via mitigating ones (e.g., van Doorn et al., 2011b), implies that over time youths become better at distinguishing between different conflict management strategies. This should be reflected in the structure of the measure. For example, conflict engagement, the most used conflict management strategy in childhood, should have a clear structure in young adolescents. On the other hand, disengagement strategies such as withdrawal and compliance might overlap during adolescence, but not in young adulthood and adulthood. Disengagement from conflict appears later on (Laursen et al., 1998, 2001), and it is likely that young adolescents might not differentiate between these strategies as well as young adults and adults do. Moreover, youths use different conflict management strategies in involuntary relationships, that is, relationships with parents and siblings, than in voluntary relationships, that is, relationships with romantic partners and best friends (e.g., Adams and Laursen, 2001). One would expect that the structure of conflict management would also differ across relationships. For example, as positive problem solving and withdrawal tend to be used more in peer relationships as opposed to families (Laursen et al., 2001), these dimensions should be better differentiated within the peer relationships and be less separated within the family. The current study examined whether the structure of conflict management is *homogeneous*, that is, whether the structure of conflict management remains the same across age and relationships, or *heterogeneous*, that is, whether the structure of conflict management differs across age groups and type of relationship.

Homogeneity vs. heterogeneity refers to different aspects. One is developmental homogeneity vs. heterogeneity, meaning that the structure of dimensions might be different for different age groups. Another aspect is heterogeneity vs. homogeneity stemming from relationships, meaning that, as relationships differ in terms of equality and voluntary character (see Adams and Laursen, 2001), the structure of conflict management strategies might be influenced by the characteristics of the relationship.

### 1.3 Conflict management, quality of relationships and adjustment

In order to validate the structure of conflict management, we examine associations of conflict management dimensions with quality of personal relationships and psychosocial adjustment.

Interpersonal conflict and conflict management are intrinsically linked to the quality of relationships. While some level of conflict is present in all relationships, the way conflicts are managed and their effect on adjustment are influenced by the quality of the relationship (Adams and Laursen, 2007; Branje, 2020). Poor quality relationships, that is relationships that are low on support and tolerance and high on negative interactions, are characterized by higher levels of conflict and by the use of coercive conflict management strategies (e.g., Missotten et al., 2017, 2018). Supportive relationships within the family were linked to more frequent use of negotiation, while poor quality relationships within the family were linked to undifferentiated use of conflict management strategies (García-Ruiz et al., 2013) or to increased use of conflict engagement (van Doorn et al., 2007). Overall, the quality of the relationships in which youth and adults are involved is reflected in the way they manage their conflicts.

The way conflicts are managed is also linked to individual adjustment, both in youth and in adults. Youth using negative conflict management strategies based on conflict engagement and withdrawal in conflicts with parents were more likely to report higher levels of internalizing and, respectively, externalizing problems (Branje et al., 2009). When both parents and youths use conflict engagement to solve conflicts in the parent-child dyad, youths were more likely to report higher levels of delinquency (van Doorn et al., 2008). Moreover, high levels of conflict and the use of coercive conflict management often are linked to relationship dissolution, especially with peers (friends, Laursen, 1993; couples, Kurdek, 1994) and to violence in romantic relationships, both as perpetrator and as victim (e.g., Bonache et al., 2016). The use of negative conflict management is thus linked to maladjustment. Drawing on coercion theory (see Patterson, 1982), the relationship between conflict management and adjustment is, most likely, bidirectional over time. Similar to how a parent with poor parental practices and a child with problematic behavior enter in a vicious cycle of reinforcement of coercion, leading to more externalizing problems in children over time (see Patterson, 1982), negative conflict management and poor adjustment are also likely to trigger and reinforce each other over time.

### 1.4 Current study

In the current study, we performed an in-depth exploration of conflict management as measured by the Conflict Resolution Styles Inventory (CRSI, Kurdek, 1994), one of the most used measures of conflict management including four conflict management strategies: *positive problem solving*; *conflict engagement*; *withdrawal*; and *compliance*. We explored whether this four-dimension structure offers the most accurate representation of the construct of conflict management, or if a unidimensional structure is more appropriate. We explored the structure of conflict management in both adolescents and young adults and adults, and both in vertical and in horizontal relationships. We followed each member of the following dyads yearly over a period of 6 years: adolescent – mother, adolescent – father, adolescent – sibling, adolescent – best friend, and mother – father, and we followed the former adolescents now young adults and their romantic partners biannually over a period of 5 years. We examined the source of common variance via second-order and bi-factor analyses. Our aim was to examine whether the variance is accounted for by a second order factor, or by an underlying general factor.

To validate the resulting conflict management structure, we used a set of auxiliary variables. Auxiliary variables related to theory are important both for understanding the general factor, and for establishing the uniqueness of specific dimensions (Reise, 2012). We have included auxiliary variables such as internalizing and externalizing problems, and aspects of relationship quality, as studies showed they are associated to conflict management over relationships and time (Branje et al., 2009; van Doorn et al., 2011a; Yu et al., 2014; Boersma-van Dam et al., 2017; Bonache et al., 2017; Missotten et al., 2017, 2018). Altogether, we used auxiliary variables informed by theory to explain the common source of variance of the conflict management construct.

### 1.5 Hypotheses

Regarding our hypotheses, first, we hypothesized that there is a general factor of conflict management accounting for the commonalities between the four factors posited by Kurdek (1994) (see Eid et al., 2017, 2018 for information on bi-factor models with reference domain). Based on previous literature and the theory of conflict management, such general factor might reflect disengagement in conflict management (see Laursen, 1993; Kurdek, 1995; D'Zurilla et al., 2004; Branje et al., 2009; Yu et al., 2014). Second, we expected more cognitively complex conflict management strategies such as negotiation, withdrawal and compliance to become clearer and more defined from adolescence to adulthood. We expected differences in the clarity of the structure of conflict management across relationships. We expected dimensions such as withdrawal and compliance to overlap in early adolescence.

## 2 Method

### 2.1 Participants

Participants came from the Research on Adolescent Development and Relationships project (RADAR) – Young (Branje and Meeus, 2018). The current study used 497 adolescents and their parents, siblings, best friends and romantic partners. We used assessments of the relationships between adolescents and their significant others for a period of 11 years, as follows: the target adolescents were assessed yearly for 6 years (Wave 1 to Wave 6), and biannually for the three other waves (Wave 7 to Wave 9), the parents, siblings and best friends were assessed yearly for 6 years (Wave 1 to Wave 6), intimate partners were assessed biannually for three waves (Wave 7 to Wave 9).

At Wave 1, the target adolescents (57% boys) were on average 13.03 years old (SD = 0.46, range 11–15 years old), their mothers were 44.40 years old (SD = 4.45, range 31–64 years old), their fathers were 46.74 years old (SD = 5.10, range 33–68 years old), their siblings (45% boys) were 14.75 years old (SD = 3.11, range 7–23 years old), and their best friends were 13.17 years old (SD = 0.78, range 11–19 years old) (see Table 1). Most of the best friends (>90% from Wave 1 to Wave 6) were the same sex as the target adolescent. At Wave 7, one third of the participants reported having a romantic partner. For most of the participants (98%), this was an opposite sex partner. On average romantic partners were 20.58 years old at Wave 7 (SD = 2.84, range 15–32 years old) (see Table 1). All target adolescents were of Dutch origin. Participants in the study came from the western and central region of the Netherlands. Almost all target adolescents (86.1%) came from intact families. Almost 40% of the mothers and 51% of the fathers had higher education, and 10.8% of the families reported a low socio-economic status. Overall, the socio-economic status of the sample was slightly higher than in the general population, in which 23% of the women with children and, respectively, 34% of the men with children have upper education (Fakkel et al., 2020).

**Table 1:** Sample description.

	**Wave 1**	**Wave 2**	**Wave 3**	**Wave 4**	**Wave 5**	**Wave 6**	**Wave 7**	**Wave 8**	**Wave 9**
**Youths**									
Age (M, range)	13.03 (11–15)	14.03 (12–16)	15.03 (13–17)	16.03 (14–18)	17.03 (15–19)	18.03 (16–20)	19.87 (18–22)	21.66 (19–24)	23.79 (22–26)
Sex (% boys)	57%	–	–	–	–	–	–	–	–
Participation	99.6%	93.8%	90.7%	87.9%	84.7%	85.7%	77.3%	73.4%	74%
**Siblings**									
Age (M, range)	14.75 (7–23)	15.73 (8–24)	16.70 (9–25)	17.72 (10–27)	18.70 (11–28)	19.67 (12–28)	–	–	–
Sex (% boys)	45.2%	44.8%	44%	44.2%	45.4%	45.9%	–	–	–
Participation	83.9%	80.5%	78.9%	76.9%	73.8%	72.4%	–	–	–
**Best friend**									
Age (M, range)	13.17 (11–19)	14.13 (12–20)	15.14 (12–21)	16.11 (13–23)	17.14 (14–28)	18.14 (15–25)	–	–	–
Sex (% boys)	54.4%	56.4%	56.4%	56.6%	57.4%	58.2%	–	–	–
Participation	90.3%	84.7%	84.3%	83.3%	79.5%	74.4%			
**Mothers**									
Age (M, range)	44.40 (31–64)	44.50 (32–65)	46.40 (33–66)	47.40 (34–67)	48.40 (35–68)	49.40 (36–69)	–	–	–
SES (Low)	27.8	–	–	–	–	–	–	–	–
SES (high)	36.7	–	–	–	–	–	–	–	–
Participation	99.6%	93%	91.3%	88.3%	84.7%	84.5%	–	–	–
**Fathers**									
Age (M, range)	46.74 (33–68)	47.68 (34–69)	48.69 (35–70)	49.69 (36–71)	50.75 (37–73)	51.68 (38–73)	–	–	–
SES (Low)	14.8	–	–	–	–	–	–	–	–
SES (high)	53%	–	–	–	–	–	–	–	–
Participation	89.7%	85.3%	83.5%	80.3%	76.1%	75.5%	–	–	–
**Romantic partners**									
Age (M, range)	–	–	–	–	–	–	20.58 (15–32)	22.36 (17–35)	24.26 (19–43)
Sex (% men)	–	–	–	–	–	–	52.3%	–	–
Participation	–	–	–	–	–	–	30.8%	33.1%	36.8%

M, Mean; SES, Socioeconomic status.

### 2.2 Procedure

The target adolescents were recruited when they were in the 6th grade. They were youths who had a sibling older than 10 years, and whose mother and father could participate in the study. The families received written information regarding the project and have given their informed consent to participate in the study prior to the data collection. Trained research assistants collected the data during yearly home visits for six consecutive years. From Wave 7, only adolescents and their romantic partners were assessed biannually for three waves. Adolescents, parents, siblings, friends, and partners filled-in questionnaires regarding their adjustment, personal characteristics, and the quality of their relationships. For each wave, each participant received a small monetary reward (i.e., 20 Euro). The project was approved by the university ethics committee.

#### 2.2.1 Attrition analysis

Detailed information about the sample selection for the RADAR project can be found elsewhere (Branje and Meeus, 2018). In the current study, of the initial sample of 497 target adolescents, 426 adolescents (85.7%) participated at Wave 6, and 369 (74%) participated at Wave 9. At Wave 9, 191 (36.8%) intimate partners of target adolescents participated. At Wave 6, 420 mothers (84.5%) and 375 fathers (75.5%) participated. At Wave 6 367 (72.4%) siblings and 395 best friends (74.4%) answered the questionnaires (see Table 1). There were no significant differences between the youth who participated in the study and those who dropped out from the study [*X*^2^ (42, 497) = 51.23, *p* = 0.155] in terms of age, conflict management strategies, internalizing problems such as anxiety, depression, and externalizing problems, all assessed at time 1.

### 2.3 Measures

#### 2.3.1 Main variables

##### 2.3.1.1 Conflict management strategies

To assess participants' conflict management strategies, we used an adapted version of the Conflict Resolution Style Inventory (CRSI, Kurdek, 1994; see van Doorn et al., 2008). Adolescents reported on their conflict management with mothers, fathers, best friends, and romantic partners. Both mothers and fathers reported on their conflict management with each other, and with the target adolescents. Siblings reported on their conflict management with mothers, and best friends and romantic partners reported on their conflict management with the target adolescents. Participants were instructed to rate on a 5-point scale ranging from 1 (“never”) to 5 (“always”) how frequently they used each of the 20 items to deal with arguments or disagreements with their child/partner/friend/mother/father. Each dimension was assessed with five items. Sample items are: “sitting down and discussing the differences of opinion” and “negotiating and trying to find a solution that is mutually acceptable” for positive problem solving; “throwing insults and digs”; “getting carried away and saying things that aren't meant” for conflict engagement; “withdrawing, acting distant and not interested”; “tuning the other person out” for withdrawal; and “let him/her have his/her own way”; “not defending my position” for compliance. Across reporters, waves and dimensions, the internal consistency (Cronbach's alpha) ranged between 0.63 and 0.88.

#### 2.3.2 Auxiliary variables

##### 2.3.2.1 Conflict frequency

To measure youths' and their parents', friends', and romantic partners' frequency of conflict, we used the Interpersonal Conflict Questionnaire (ICQ, Laursen, 1993). The dyads reported on the level of conflict with each other. Youths, parents, friends, siblings and romantic partners answered 35 items tapping how often they had conflicts with each other during the last week over a range of conflict topics. Examples of items are: “Criticisms, teasing, put-downs” and “Differences of idea or opinion (e.g., about politics, plans about the future).” Answers ranged from 1 (“never”) to 5 (“often”). Cronbach's alpha across reporter and wave ranged from 0.81 and 0.87.

##### 2.3.2.2 Relationship quality

We used the Network Relationship Inventory (Furman and Buhrmester, 1985) to measure participants' quality of relationship with each other. The Network Relationship Inventory taps mothers', fathers', adolescents', friends', and partners' *perception of support, power assertion*, and *negative interactions* with each other. The instrument has 24 items, and answers range from 1 (“not at all”) to 5 (“a lot”). An example of item is: “How much does your best friend appreciate the things you do?” Cronbach's alpha across measures, reporters and waves ranged from 0.80 and 0.95.

##### 2.3.2.3 Adjustment measures

To assess youths' and parents' *externalizing* and *internalizing problems*, we combined four scales adapted to Dutch population. For *externalizing problems* in youths and adults, we used the Youth Self-Report scale (YSR, Achenbach, 1991) for target adolescent, best-friend, and sibling, and the Adult Self-Report scale (ASR, Achenbach and Rescorla, 2003) for mothers and fathers, and for target adolescent and intimate partner from Wave 7 onwards. For measuring target adolescent, best friend, sibling, and intimate partner *internalizing problems*, we used the Screen for Child Anxiety Related Emotional Disorders (SCARED, Birmaher et al., 1997) and the Reynolds Adolescent Depression Scale- 2nd edition (RADS-2, Reynolds, 2004). We used the Adult Self-Report to measure internalizing problems in adults. Cronbach's alpha ranged between 0.58 and 0.91 across measures, reporters and waves. We combined the measures of adjustment in two latent variables: an externalizing variable, and an internalizing variable. To measure *prosocial behavior*, we used the score for the prosocial scale from the Dutch adaptation of the Self-report of Aggression and Social Behavior Measure (Morales and Crick, 1998).

### 2.4 Strategy of analysis

#### 2.4.1 Data preparation for analysis

As for Wave 1, a subgroup of more than half of the target adolescents received a shorter questionnaire, in which two out of five items per conflict resolution scale were removed, there was a high percentage of missing data for this wave by research design. For all the other five waves (Wave 2 – Wave 6) for target adolescents and for all other respondents (Wave 1 – Wave 6 for mothers, fathers and friends and Wave 7 – Wave 9 for youths and their romantic partners), between 0.4 and 10% of data was missing per item. We checked the pattern of missingness in our data wave by wave, and by respondent. Little's MCAR tests (Little, 1988) for each wave and respondent were significant in less than half of the analyses, and when they were significant, using Bollen's Chi-square adjustment for sample size (Bollen, 1989), most of the *X*^2^*/df* ratios were under 2:1. This means that we can use imputation to account for missing data. Incidental missing values at item level were imputed using the Expectation Maximization (EM, Dempster et al., 1977) algorithm in SPSS (Meeus and Branje, 2018). We used Full Information Maximum Likelihood (FIML, Enders and Bandalos, 2001) for handling the remaining missing data in Mplus (Muthen and Muthen, 1998–2019).

#### 2.4.2 First-order factor analysis

As our aim was to understand the underlying structure of the conflict management construct, we used an approach with models increasing in complexity (Reise, 2012; Rodriguez et al., 2016). First-order models are a necessary step before fitting any higher-order models, as they allow to test three necessary assumptions: (1) A good fitting CFA model as a base for the higher-order models; (2) Significant correlations between the first order factors that could justify the use of second-order and bifactor modes (Brown, 2015); and (3) No redundancy between the first-order dimensions (Reise, 2012).

**Figure 1:**
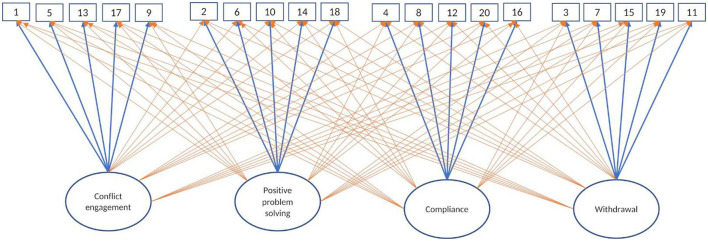
Nested Confirmatory Factor Analysis within Exploratory Structural Equation Modeling Note. Model 1: (a) Confirmatory Factor Analysis (CFA) nested within a (b) Exploratory Structural Equation Modeling (ESEM). The darker and bolded arrows represent the CFA model (Model 1a), the lighter and thinner arrows represent the model building for CFA-ESEM (Model 1b).

As a first step, to examine whether the structure of conflict management is the same over time and across relationships, we used Exploratory Structural Equation Modeling (ESEM, Marsh et al., 2014) in addition to traditional Confirmatory Factor Analyses (CFA, Joreskog and Sorbom, 1979). We chose this approach, rather than the traditional use of Exploratory Factor Analysis (EFA) followed by a CFA, for several reasons. First, CFA-ESEM allows to perform the exploratory and confirmatory phase on the same sample, avoiding the differences in model fit between exploratory and confirmatory models in traditional factor analyses (see Brown, 2015). Second, CFA-ESEM allows items to cross-load on several dimensions, solving the model fit issues and overestimated factor correlations often encountered when using CFA (Asparouhov and Muthen, 2009). A CFA-ESEM approach helps us understand whether the correlations between the conflict management dimensions are due to item cross-loadings. Starting from the base model (Model 1a, see Figure 1) – a classic CFA model with correlated dimensions – for each respondent and wave, we then expanded to an ESEM by allowing all items to load on all dimensions (Model 1b, see Figure 1). Over and above model fit indices, a dimension is well-defined if its items loaded highly on it and loaded below 0.30 on other dimensions. We considered dimensions as poorly differentiated when more than three of their items cross-loaded on other dimensions with loadings above 0.40.

In all our analyses, we winsorized several items from the conflict engagement dimension, as they were slightly skewed (skewness > 2 and kurtosis > 5). We limited the extreme values to the highest threshold, that is to 3, to reduce bias. We winsorized the following items: item 1 and item 17 for youths' reports on their conflict management strategies with mothers and fathers, and for friends' reports; item 1, 5, 13, and 17 for youths' reports on conflict management with friends; and item 17 for parents' reports on conflict management with their children.

#### 2.4.3 Second-order factor analysis

Provided that a fundamental condition that justifies the use of higher-order/ bifactor models was fulfilled, that is, that our first-order dimensions are correlated (see Brown, 2015), we proceeded with higher-order and bifactor modeling. For the exploration of the structure of conflict management, we used both a second-order factor analysis and a bifactor analysis. For the second-order factor and bifactor models, we grouped our analyses based on type of reporting. Target adolescent and sibling reports on conflict management with parents were analyzed together. Fathers' and mothers' reports on conflict management with target adolescents were analyzed together, and husbands' and wives' reports on the way they manage conflict in their relationship were analyzed together. Target adolescent and best friend reports on conflict management in their relationship were analyzed together, and so were the target adolescent and intimate partner reports. As the data had a multilevel structure due to this grouping, we took clustering into account when estimating all our higher-order models using the Type=Complex command in Mplus (Asparouhov and Muthén, 2006).

As the data had a multilevel structure, we tested for dependency of scores within relationships (Kenny et al., 2006). As conflict management strategies differ based on participants' sex (see Laursen, 1995), we used this variable to distinguish between dyad members in romantic and marital relationships. The results showed low levels of dependency for the distinguishable dyads: The overall pattern of correlations (Pearson's r, see Kenny et al., 2006) between mother and father is inconsistent, with most correlations being small and non-significant (rs ≤ 0.20). We found the same pattern for the target adolescent – intimate partner dyad. For the non-distinguishable dyads (e.g., adolescent and best friend), though, Intra-Class Correlations (ICCs, Hox, 2010) coefficients were slightly above the threshold of 0.05 (see Kenny et al., 2006). Thus, we used Type = Complex command in Mplus (Asparouhov and Muthén, 2006).

**Figure 2:**
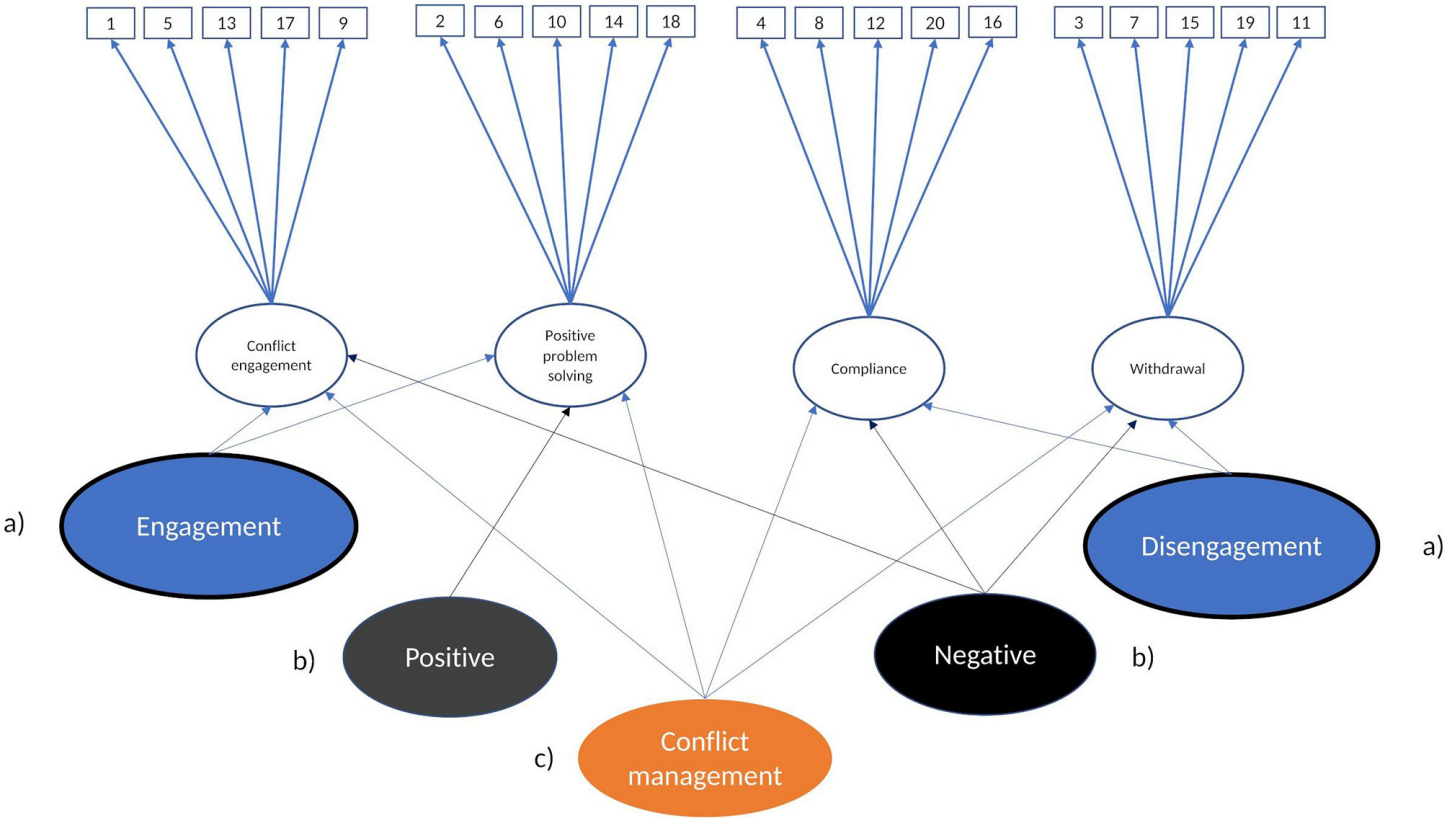
Second-order factor models. Model 2: (a) Second-order factor model for engagement, (b) Second-order factor for positivity, and (c) Second-order factor model for all dimensions.

We used the second-order factor analysis to test whether the four dimensions can be explained by two broader dimensions of conflict management posited by theory: positivity and engagement. We started with the basic CFA model with four factors (Model 1a, see Figure 1), on which we added the two second-order factors: positivity (Model 2a, see Figure 1) and engagement (Model 2b) (see Figure 2). As for Wave 2 and Wave 3 youths did not report on the Withdrawal scale, we left these waves out of our analyses for youths' reports. In order to identify the higher-order part of the model, all second-order factor loadings were constrained to be equal (Brown, 2015).

#### 2.4.4 Bifactor analysis

**Figure 3:**
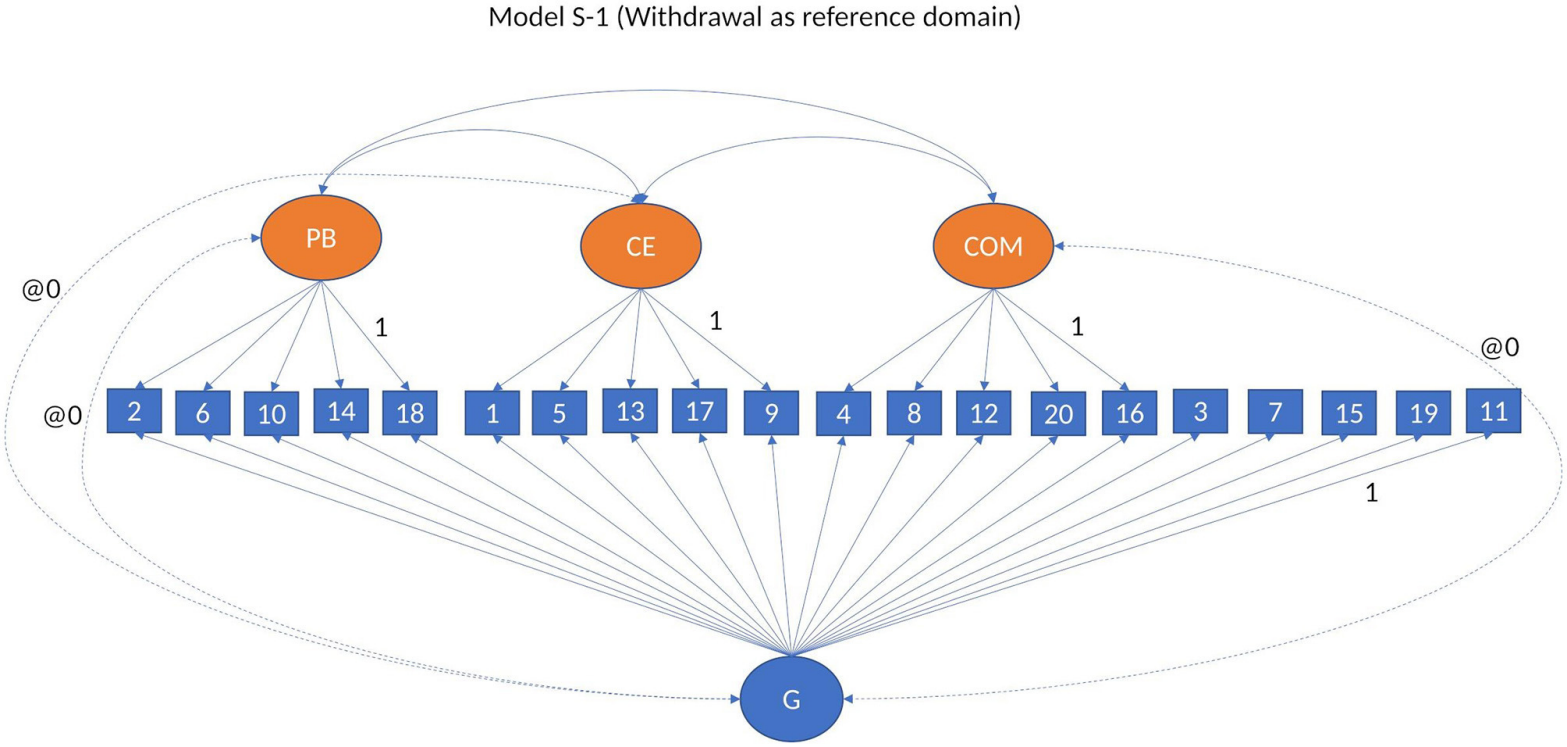
Bifactor model S-1. Model 3: Modified bifactor model withdrawal as reference domain.

To test whether there is a general factor of conflict management that accounts for the commonalities between the items, in addition to the specific dimensions, we built a bifactor model with reference domain starting from the basic CFA model. In this model (Model 3, S-1, see Figure 3), all items were allowed to load on a general factor of conflict management, as well as their regular loading on the specific dimensions, except for the items of the reference domain, in this case withdrawal, which load only on the general factor. A bifactor model tests the multidimensionality of conflict management, while accounting for the commonality of all observed variables. As theoretically the dimensions of conflict management cannot be seen as interchangeable, we fitted models in which withdrawal was used as a reference domain for our general factor (Model 3, S-1, Eid et al., 2017, 2018), and in which the specific factors were allowed to correlate. In all models, the correlations between the general factor and the separate dimensions are set to zero.

#### 2.4.5 Validation of the measure

**Figure 4:**
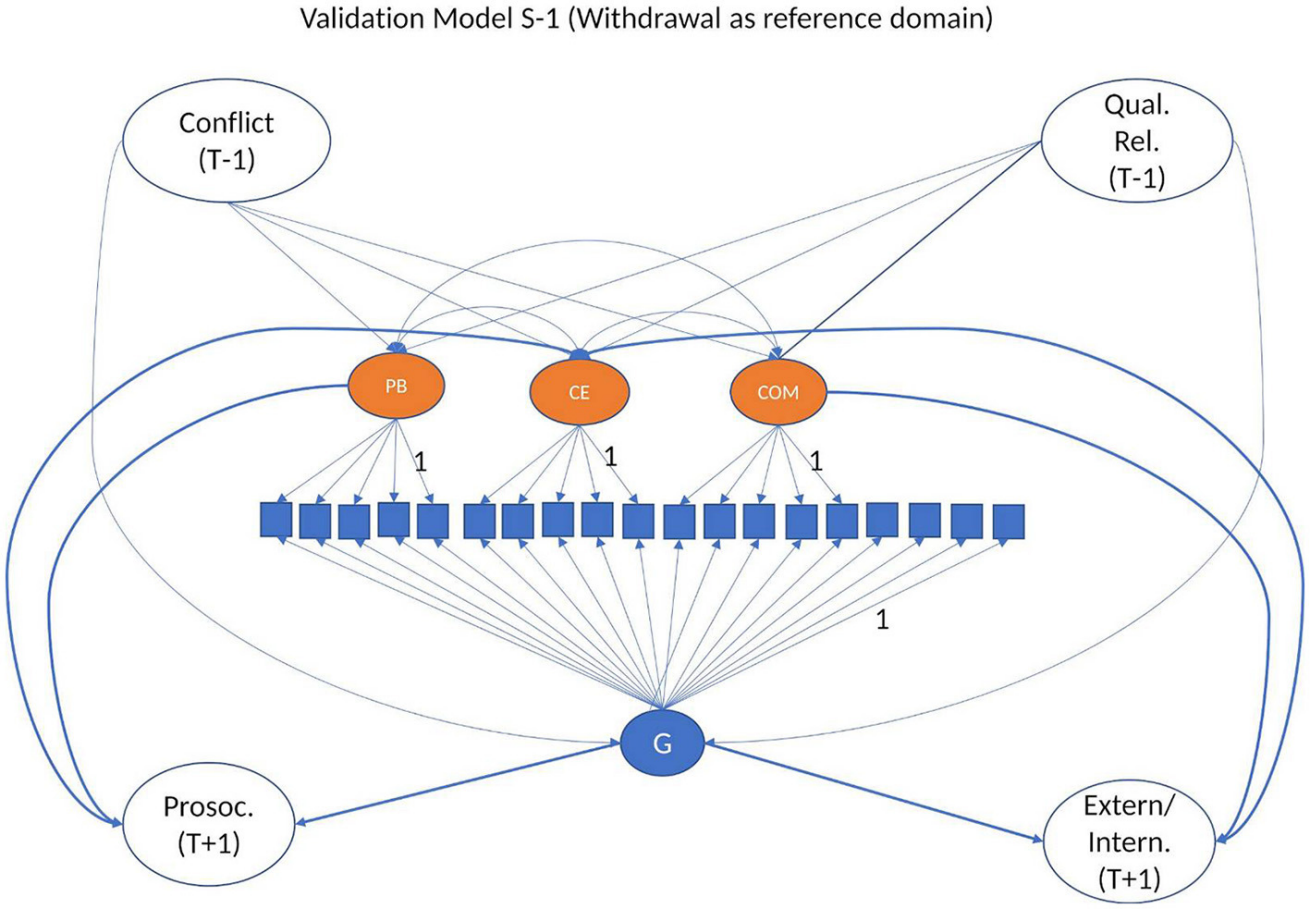
Validation Model for S-1 Bifactor Model with Withdrawal as Reference Domain. Model 4: (a) and (b) Bifactor model with quality of relationship and conflict as predictors of G and S factors; (c) Bifactor model with G and S factors as predictors of externalizing and internalizing problems; (d) Bifactor model with G and S factors as predictors of prosocial behavior.

To understand the origin of the variance behind the structure of conflict management, we used conflict intensity and relationship quality as predictors (W-1) (see Model 4a and Model 4b, Figure 4), and adjustment problems and prosocial behaviors as outcomes (W+1) of our dimensions (Model 4c and Model 4d, see Figure 4). The base for these validation models is bifactor models with reference domain (Model 3, S-1, see Figure 3) as they are suited for predicting real-world criteria (see Eid et al., 2018). The general factor in these models is conflict management as captured by its reference domain withdrawal.

#### 2.4.6 Model fit evaluation

The model fit was evaluated using the following fit indices: Comparative Fit Index (CFI, Bentler, 1990), the Tucker-Lewis Index (TLI, Bentler and Bonett, 1980), the Root Mean Square Error of Approximation (RMSEA, Steiger, 1990), and the Standardized Root Mean Square Residual (SRMR, Hu and Bentler, 1999). A CFI and TLI index around 0.95, and a RMSEA and SRMR smaller than 0.08 are typically indicators of a good model fit (Hu and Bentler, 1999). In the models accounting for nestedness, Maximum Likelihood Robust (MLR, Chou et al., 1991; for Mplus 7, see Muthen and Muthen, 1998–2019) was used as estimator. We selected the best-fitting model not only based on tests of model fit, but also based on theoretical relevance and parsimony.

In the bifactor models, to evaluate whether the specific factors are self-standing, aside from their commonalities captured by the general factor, we used the following estimates: the Factor Determinacy Index (FDI, Beauducel, 2011), the Explained Common Variance (ECV, Rodriguez et al., 2016), Omega/OmegaH (internal reliability of latent variable, Rodriguez et al., 2016) and the H-index (Hancock and Mueller, 2001). For FDI, values of 0.80 and above are considered adequate for dimensions to be considered reliable. An H-index equal to or larger than 0.70 indicates how much of the factor variance is accounted for by its indicators (Hancock and Mueller, 2001). An ECV range of 0.70–0.85 indicates that most of the common variance is due to the general factor, thus implying that a measure is unidimensional (Rodriguez et al., 2016).

## 3 Results

Due to the many reporters and waves, we reported only ranges of means, and standard deviations for the conflict management scales across reporters (see Table 2). Detailed descriptive statistics for individual items are available upon request from the first author.

**Table 2:** Ranges of item means and standard deviations per type of respondent for the scales of the Conflict Resolution Styles Inventory.

**Scale/Type of relationship**	**Conflict engagement**	**Positive problem solving**	**Withdrawal**	**Compliance**
**Adolescent and best friend reporting on relationship with each other**				
Mean	1.22–1.75	2.87–3.63	1.55–1.96	1.42–2.38
SD	0.46–0.91	1.03–1.17	0.75–0.99	0.74–0.99
**Adolescents reporting on relationship with parents**				
Mean	1.32–2.06	2.80–3.26	1.89–2.30	1.71–2.56
SD	0.62–1.02	0.96–1.10	0.91–1.02	0.67–0.98
**Parents reporting on relationship with adolescents**				
Mean	1.21–2.11	3.62–3.98	1.67–1.86	1.71–2.32
SD	0.41–0.90	0.66–0.85	0.77–0.88	0.70–0.95
**Parents reporting on relationship with each other**				
Mean	1.21–2.10	3.60–3.92	1.85–2.31	1.86–2.56
SD	0.47–0.97	0.69–0.87	0.82–1.00	0.76–0.96
**Youths and romantic partner reporting on relationship with each other**				
Mean	1.20–2.10	3.60–3.95	1.73–2.42	1.59–2.49
SD	0.50–1.02	0.79–0.93	0.81–1.05	0.76–0.95

SD, Standard deviation; W, Wave.

### 3.1 The dimensionality of conflict management from early adolescence to adulthood

In order to be able to apply the higher-order models, we first needed to test several assumptions (Reise, 2012; see Brown, 2015). A first assumption is that the CFA model on which the higher-order models are based needs to have an acceptable fit. Stemming from the first assumption is another assumption: the commonalities between the dimensions should not be accounted for by dimension redundancy.

In order to test the assumption that the model on which the higher-order models are based has an acceptable fit, we fitted sixty CFA models with correlated dimensions for each respondent, relationship, and wave (see Model 1a, Figure 1). As our measure includes both positively and negatively worded items, we allowed several correlated errors to account for measurement effects (see Brown, 2015). This assumption was met: The model fit ranged between acceptable [i.e., CFI/TLI = 0.91/0.90, RMSEA = 0.070 (0.062–0.077), SRMS = 0.059] and very good [i.e., CFI/TLI = 0.97/0.97, RMSEA = 0.042 (0.031–0.052), SRMS = 0.038] (see Supplementary Table 1). The correlations between dimensions such as conflict engagement, compliance and withdrawal were often r ≥ 0.60 (in 52% of the models), and r ≥ 0.50 (in 81% of the models) in these models, which can be due to overlap between the dimensions.

In order to test the third assumption, that the conflict management dimensions are distinct and can be considered independent without item cross-loadings, we fitted the sixty CFA - ESEM models via removing the CFA restrictions and allowing items to cross-load on all four dimensions (see Model 1b). The model fit ranged between good [i.e., CFI/TLI = 0.95/0.92, RMSEA = 0.064 (0.055–0.073), SRMS = 0.029] and excellent [i.e., CFI/TLI = 0.99/0.98, RMSEA = 0.030 (0.000–0.050), SRMS = 0.029] (see Supplementary Table 2). Few items cross-loaded significantly across respondents and relationship type (βs ranging between 0.14 and 0.41 across waves and respondents). These cross-loadings did not affect the item loading on their respective dimension. Despite the items being allowed to load on all dimensions, the dimensions still correlated significantly. Withdrawal continued to correlate average to moderate (*rs* between 0.30 and 0.65) with both conflict engagement and compliance. This means that the item cross-loadings cannot account for the shared variance between dimensions. Thus, for all our higher-order analyses, we used the four-factor CFA model as a base (i.e., Model 1a, Figure 1).

The results of the CFA and CFA-ESEM models confirmed that the conflict management construct has four correlated dimensions across respondents and waves: Except for about 5% of items with sporadic lower factor loadings, often close to 0.40, all items loaded above 0.40 on their corresponding dimensions. Our results showed that the structure was similar across time and type of relationship. Correlations were larger when youths were the respondent, and when they reported on their relationship with peers. As the shared variance between the four dimensions is not accounted for by the dimensions overlapping with each other, something else might account for the common variance between these dimensions.

### 3.2 The higher-order structure of conflict management across age

In order to examine whether the construct of conflict management is unidimensional or multidimensional, and what explains the shared variance between the four dimensions of conflict management, for each type of relationship, we fitted two types of higher-order factor models: second-order factor models (Model 2a and Model 2b) (see Figure 2), and bi-factor models (Model 3, S-1) (see Figure 3). In these models, we grouped our analyses based on the type of reporting.

#### 3.2.1 Second-order models

We first fitted two second order models derived from theory: one model for positivity (Model 2a), and one model for engagement (Model 2b) (see Figure 2). Overall, we fitted 23 models for each theoretical model. The overall model fit of the second-order models was poor for 83% of the positivity models and for 100% of the engagement models (see Supplementary Table 4). Thus, the second-order dimensions of conflict management were not supported by the data.

For the second order factor model in which all dimensions were allowed to load on a single second order factor (Model 2c, Figure 2), more than 17% had a poor fit (see Supplementary Table 5). We used this model as a parsimonious higher-order factor model compared with the bifactor model, in order to ascertain whether conflict management construct is unidimensional or multidimensional.

#### 3.2.2 Bifactor models

Our next step was to fit bifactor models to test whether the shared variance between the dimensions of conflict management can be explained by a general factor of conflict management. We used a bifactor model with withdrawal as reference domain (Model 3, S-1, Figure 3) (see Supplementary Table 6). The model fit of the bifactor model with withdrawal as reference domain was good across respondent type and waves [i.e., between CFI/TLI = 0.92/0.90, RMSEA = 0.059 (0.055–0.065), SRMS = 0.050 and CFI/TLI = 0.96/0.95, RMSEA = 0.040 (0.034–0.045), SRMS = 0.035]. We used this model (i.e., Model 3, S-1) as base for all our bifactor analyses.

Across waves and type of reporting, all items loaded significantly and, with very few exceptions, adequately on their *specific dimensions* (i.e., positive problem solving, conflict engagement and compliance). Standardized factor loadings (βs) ranged between 0.32 and 0.88 for *positive problem* solving, between 0.32 and 0.69 for *conflict engagement*, and between 0.23 and 70 for *compliance* (see Table 3; for more details, see Supplementary Table 7). The Factor determinacy index (FDI) ranged between 0.90 and 0.95 for positive problem solving, between 0.79 and 0.88 for conflict engagement, and between 0.73 and 0.88 for compliance. All specific factors had significant variance. ECV for specific factors ranged between 0.70 and 0.95 for posi*tive problem solving*, between 0.58 and 0.78 for *conflict engagement*, and between 0.58 and 0.89 for *compliance*. The H-index ranged between 0.80 and 0.91 for *positive problem solving*, between 0.60 and 0.74 for *conflict engagement*, and between 0.51 and 0.77 for *compliance*. Omega ranged between 0.80 and 0.90 for *positive problem solving*, between 0.77 and 0.83 for *conflict engagement*, and between 0.61 and 0.82 for *compliance*. This means that the specific dimensions are, overall, well-defined, and non-collapsible into a unidimensional construct.

**Table 3:** Standardized factor loadings for the general and specific factors in the S-1 Bifactor models.

	**Estimates for adolescents**		**Estimates for adults**	
**Item**	β	**S.E**.	β	**S.E**.
**General factor**				
1. Personally attack him/her	0.24–0.34	0.034–0.044	0.25–0.35	0.037–0.066
2. Focusing on the problem at hand	−0.14–0.02	0.037–0.052	−0.12–−0.38	0.035–0.073
3. Remaining silent for long periods of time	0.33–0.63	0.025–0.051	0.51–0.68	0.024–0.059
4. ^*^Not being willing to stick up for myself	0.16–0.43	0.034–0.042	0.22–0.52	0.037–0.064
5. Exploding and getting out of control	0.36–0.51	0.032–0.037	0.27–0.38	0.035–0.063
6. Sitting down and discussing differences constructively	−0.09–−0.29	0.034–0.039	−0.30–−0.53	0.032–0.071
7. Reaching a limit, “shutting down,” and refusing to talk any further	0.50–0.72	0.022–0.037	0.63–0.80	0.018–0.048
8. Being too compliant	0.15–0.45	0.034–0.047	0.11–0.41	0.036–0.065
9. Getting carried away and saying things that aren't meant	0.43–0.62	0.031–0.037	0.37–0.44	0.034–0.058
10. Finding alternatives that are acceptable to each of us	−0.02–−0.31	0.035–0.039	−0.27–−0.45	0.033–0.077
11. Tuning the other person out	0.70–0.80	0.021–0.032	0.62–0.80	0.020–0.047
12. ^*^Not defending my position	0.20–0.46	0.032–0.043	0.24–0.51	0.036–0.070
13. Throwing insults and digs	0.39–0.52	0.031–0.037	0.30–0.55	0.034–0.056
14. Negotiating and compromising	−0.04 to −0.26	0.034–0.038	−0.14–−0.42	0.036–0.074
15. Withdrawing, acting distant and not interested	0.69–0.79	0.018–0.032	0.67–0.81	0.019–0.041
16. Giving in with little attempt to present my side of the issue	0.24–0.44	0.033–0.049	0.27–0.45	0.034–0.062
17. Getting so angry that I do not know what I am doing anymore	0.35–0.51	0.030–0.043	0.25–0.41	0.034–0.063
18. Searching for a solution that is good for both of us	−0.06–−0.34	0.035–0.045	−0.25–−0.43	0.034–0.079
19. ^*^Not responding to him/her anymore	0.74–0.85	0.016–0.036	0.72–84	0.016–0.038
20. Let him/her have his/her own way	0.14–0.41	0.033–0.049	0.10–0.32	0.036–0.067
**Conflict engagement**				
1. Personally attack him/her	0.36–0.59	0.042–0.080	0.36–0.67	0.029–0.076
5. Exploding and getting out of control	0.48–0.61	0.037–0.080	0.56–0.69	0.032–0.067
9. Getting carried away and saying things that aren't meant	0.32–0.68	0.035–0.081	0.60–0.71	0.034–0.066
13. Throwing insults and digs	0.41–0.56	0.037–0.069	0.35–0.64	0.033–0.063
17. Getting so angry that I do not know what I am doing anymore	0.38–0.61	0.043–0.088	0.36–0.53	0.035–0.083
**Positive problem solving**				
2. Focusing on the problem at hand	0.39–0.65	0.030–0.044	0.33–0.52	0.036–0.080
6. Sitting down and discussing differences constructively	0.59–0.74	0.021–0.030	0.46–0.65	0.031–0.070
10. Finding alternatives that are acceptable to each of us	0.78–0.86	0.017–0.024	0.68–0.83	0.025–0.050
14. Negotiating and compromising	0.73–0.84	0.017–0.025	0.58–0.74	0.029–0.065
18. Searching for a solution that is good for both of us	0.82–0.88	0.015–0.029	0.76–0.85	0.022–0.044
**Compliance**				
4. ^*^Not being willing to stick up for myself	0.31–0.54	0.034–0.055	0.28–0.63	0.042–0.085
8. Being too compliant	0.54–0.68	0.034–0.063	0.40–0.70	0.042–0.078
12. ^*^Not defending my position	0.35–0.53	0.037–0.065	0.26–0.63	0.041–0.081
16. Giving in with little attempt to present my side of the issue	0.43–0.60	0.033–0.059	0.53–0.69	0.042–0.085
20. Let him/her have his/her own way	0.47–0.55	0.035–0.059	0.37–0.60	0.043–0.082

^*^Items coded on the reverse. More than 99% of the factor loadings on the general factor in adults were significant. More than 93% of the factor loadings on the general factor in adolescents were significant. All the non-significant factor loadings on the general factor regarded items belonging to the positive problem-solving dimension. All factor loadings for the specific factors were significant. In youths, about 50% of the factor loadings on the general factor were ≥ 0.40, and 64% of them were ≥ 0.30. In adults, about 47% of the factor loadings were ≥ 0.40, and about 80% of the factor loadings were ≥ 0.30.

**Figure 5:**
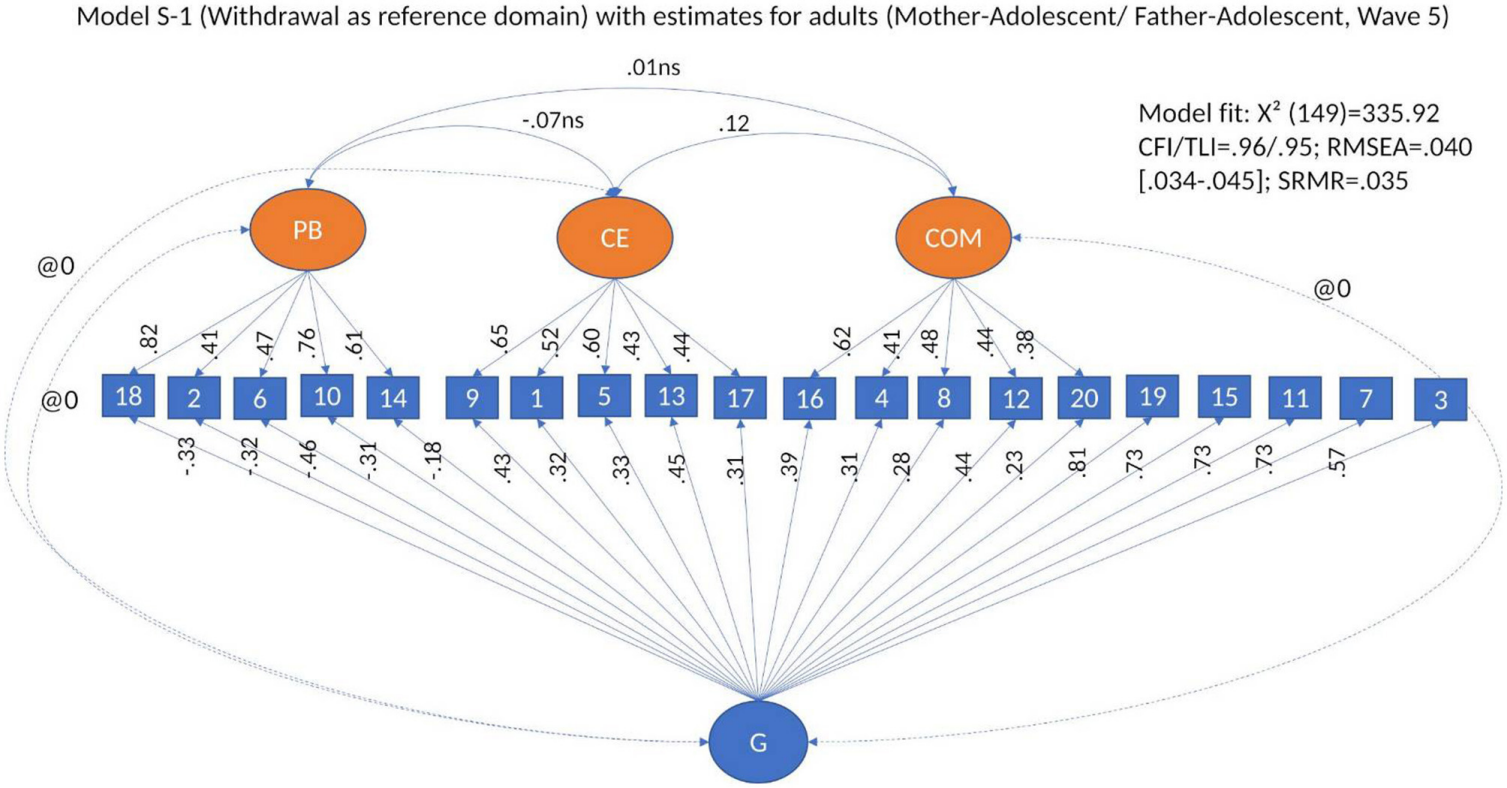
Model estimates for S-1 Bifactor Model with withdrawal as reference domain in parents' conflict management with adolescents.

**Figure 6:**
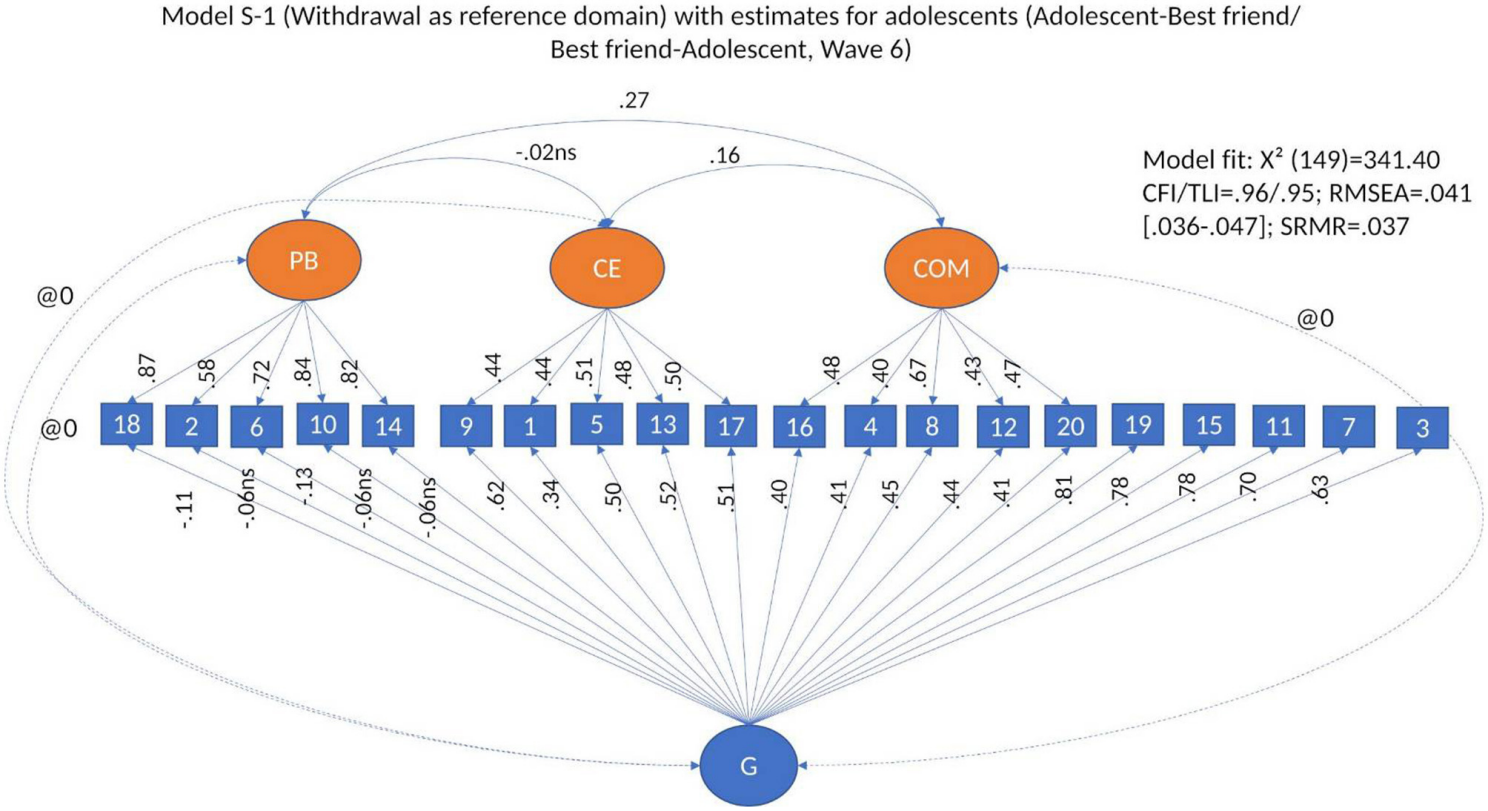
Model estimates for S-1 Bifactor Model with withdrawal as reference domain in adolescents' and best friends' conflict management.

We noted differences in the structure of the general factor by type of respondent. In models based on adult reports, such as mothers and fathers reporting on conflict management with each other and with the target adolescent, and young people reporting on their relationship with their romantic partner, all items loaded significantly on the general factor (see Supplementary Table 7 and Figure 5 for an example of model estimates in adults). Nevertheless, in models based on data in which the respondents were adolescents, that is, adolescents' reports on conflict management strategies with parents, and adolescent and best friend reporting on conflict management strategies with each other, several items from the positive problem-solving dimension consistently loaded non-significantly or had very low factor loads on the general factor (see Figure 6 for an example of model estimates in adolescents). This means that, at the bifactor level, conflict management was heterogeneous: the general factor in adults differed from the general factor in adolescents.

Across respondents and waves, for the *general factor*, standardized factor loadings (βs) ranged between −0.13 and −0.53 in adults, and between 0.02 and −0.34 in adolescents for items of the *positive problem-solving* dimension; between 0.25 and 0.55 in adults, and between 0.24 and 0.57 in adolescents for items of *conflict engagement*; between 0.10 and 0.57 in adults and between 0.14 and 0.46 in adolescents for items of *compliance*; and between 0.44 and 0.85 in adults, and between 0.33 and 0.85 in adolescents for items of *withdrawal* (see Table 3; for more details, Supplementary Table 7). All of the general factor loadings in models with adult as respondents were significant, and 93% of the loadings on the general factor were significant in adolescents. In adolescents, roughly 50% of the factor loadings on the general factor across all models were equal to or above 0.40 and 64% of factor loadings were equal to or above 0.30. In adults, 47% of factor loadings on the general factor across all models were equal to or above 0.40, and about 80% of the factor loadings were equal to or above 0.30 (see Table 3; for more details, see Supplementary Table 7). FDI for the general factor were between 0.92 and 0.95 in adult respondents, and between 0.92 and 0.94 in adolescents. ECV ranged between 0.38 and 0.51 in adult respondents and between 0.42 and 0.48 in adolescents, and H-index ranged between 0.51 and 0.92 in adult respondents, and between 0.57 and 0.91 in adolescents. Omega (i.e., internal reliability for the latent general factor) ranged between 0.78 and 0.87 in adult respondents, and between 0.79 and 0.90 in adolescents. The bifactor model was best represented in the models in which adults reported on conflict management with each other, followed by adults reporting on their adolescent, and by romantic partners reporting on conflict management with each other. This means that the structure of conflict management becomes better defined with age.

As the model fitting the data better was the bifactor model with withdrawal as reference domain (i.e., Model 3, S-1, see Figure 3), we used this model in our validation analyses.

### 3.3 Explaining the common source of variance of conflict management

In order to explain the variance in conflict management dimensions, starting from the bifactor model using withdrawal as reference domain (Model 3, S-1, see Figure 3), we fitted models in which conflict frequency and relationship quality were predictors (T-1) of conflict management dimensions, both general and specific (Model 4a and Model 4b) (see Figure 4), and the general and specific dimensions of conflict management were predictors of internalizing and externalizing problems (T+1) (Model 4c and Model 4d) (see Figure 4). We have split our auxiliary variables across several models as bifactor models are by default complex, and any new added variable takes its toll on the model fit and model convergence. Due to the complexity of the models, in order for the models to converge, the auxiliary variables were all observed variables.

All models had an acceptable [CFI/TLI = 0.91/0.89, RMSEA = 0.044 (0.038–0.049), SRMS = 0.063] to very good fit [CFI/TLI = 0.97/0.96, RMSEA = 0.029 (0.020–0.037), SRMS = 0.046]. Overall, we found small to medium effect sizes (see Supplementary Tables 8–10 for estimates). As expected, poor conflict management strategies such *as conflict engagement* and *compliance* were linked to *higher levels* of *internalizing problems* (βs between 0.03 and 0.27, 81% significant estimates). Conflict engagement was significantly linked to higher levels of *externalizing problems* one year later (βs between 0.05 and 0.38, 100% significant estimates). *Positive problem solving* was linked to *more prosocial behavior* 1 year later (βs between 0.10 and 0.25, 100% significant estimates). The *general factor* was linked to *more internalizing* and *externalizing problems* 1 year later (βs between 0.11 and 0.37, 100% significant estimates), as well as to less *prosocial behavior* (βs between −0.06 and −0.30, 87% significant estimates).

Regarding the predictors of conflict management strategies, the results supported the expectations: *higher levels of conflict* in the relationship, as well as perceived *negativity* in interactions were linked to *higher levels of conflict engagement* 1 year later (βs between 0.17 and 0.40, 100% significant estimates). Higher levels of *support* in the relationship were related to *higher levels of positive problem solving* 1 year later (βs between 0.12 and 0.34, 100% significant estimates). Higher levels of perceived *power assertion* by the other member in the relationship were related to *higher levels of compliance* 1 year later (βs between 0.15 and 0.55, 100% significant estimates). Regarding the *general factor* of conflict management, *higher levels of conflict* (βs between 0.18 and 0.32, 100% significant estimates), *negativity* (βs between 0.15 and 0.33, 100% significant estimates), and *power assertion* (βs between 0.03 and 0.18, 81% significant estimates) were linked to higher levels of this dimension, and higher levels of *support* (βs between −0.06 and −0.28, 81% significant estimates) were related to *lower* levels of general conflict management strategies.

## 4 Discussion

Conflicts and conflict management are part of daily life. The way conflicts are managed impacts youths' development (e.g., van Doorn et al., 2008; Branje et al., 2009), relationship quality (e.g., Adams and Laursen, 2007; Branje, 2020) and duration (e.g., Gottman, 1993), and adjustment in youths (e.g., delinquency, van Doorn et al., 2008; depression, Boersma-van Dam et al., 2017) and adults (e.g., couple violence, victimization, Bonache et al., 2016, 2017). Nevertheless, while some studies saw conflict management as unidimensional (Castellani et al., 2014), others explored a range of conflict management dimensions (Kurdek, 1994; Bonache et al., 2016). In our study, we explored the multidimensional vs. unidimensional structure of conflict management in family relationships, friendships and romantic relationships. We compared the structure of conflict management via first-order, second-order, and bifactor measurement models across different relationships and ages. The first-order models (CFA) showed that the four dimensions of conflict management, that is, conflict engagement, positive problem solving, withdrawal, and compliance, could be measured similarly for adolescents, young adults and adults, and across different relationships and ages. The second-order models showed no support for the theoretical higher-order dimensions positive/negative and engagement/disengagement. The bifactor models supported the multidimensionality of conflict management: partly confirming our hypotheses, there were commonalities between the four dimensions captured by the general factor, but there were also three separate contributions brought by each specific dimension. The bifactor models also showed differences between adults and young adults, and youths in the structure of conflict management: while positive problem solving was part of a general factor of conflict management in adults and young adults, it was not part of the general factor in adolescents.

Our results are in line with studies on conflict management that assert its multidimensionality. Previous studies using the same instrument as the current study have identified between three and five dimensions of conflict management (e.g., four dimensions, Kurdek, 1994; three dimensions, Bonache et al., 2016). Although these studies found repeatedly that conflict management is multidimensional (Kurdek, 1994, 1995), some studies tried to address the moderate correlations between the dimensions at first-order factor level via collapsing dimensions, which improved model fit, but sacrificed interpretability (e.g., young adults, Bonache et al., 2016). The current study aimed at solving this issue using bi-factor models, which account for the commonalities between the dimensions of conflict management, while allowing specific contributions for each dimension. Our results that the factor loadings on the general factor, while significant, were not, on average, higher than the factor loadings on the specific dimensions, supported the hypothesis of multidimensionality of conflict management. Although there are separate contributions brought by each of the specific dimensions positive problem solving, conflict engagement and compliance, there are also commonalities between the four dimensions captured by the general factor, with withdrawal as its reference domain. In terms of theory, this means that we cannot consider conflict management as a purely multidimensional construct. There are commonalities between the dimensions, in our case captured as disengagement and negative conflict management, which brings support to the theoretical assumption that conflict management dimensions are characterized by positive vs. negative and engagement vs. disengagement axes.

In line with our hypotheses, the bifactor structure with withdrawal, that is, disengagement, as reference domain for the general factor had a good fit. This means that this general factor accounts for commonalities the other dimensions have with withdrawal, and that the specific factors account for the unique contributions each dimension brings aside from their commonalities with withdrawal. Withdrawal as a dimension lost specificity, reflecting that the specific factor withdrawal disappeared into the general factor. This is not unexpected, as withdrawal in itself does not solve conflict, but it is rather a transition strategy. Moreover, withdrawal gives the general factor its meaning (Eid et al., 2018). The general factor could be seen as a collection of negative reactions of disengagement when experiencing conflict. Negative specific dimensions such as conflict engagement and compliance represent that part of the conflict engagement and compliance domains not shared with the reference domain withdrawal. And the same is valid for positive problem solving in adults. The general factor, overall, taps into the extent to which people use a maladaptive strategies when dealing with conflicts, while the specific factors tap into the use of conflict engagement, compliance and positive problem-solving that is not common with withdrawal.

A notable mention regarding bi-factor models with reference domain is that they allow for a much more complex exploration of constructs. In our study we chose withdrawal, a disengaging negative dimension, as our domain. Both the general factor and the specific factors are defined by the commonalities with this domain. This means that the general factor captures the commonalities all domains have with withdrawal, while the specific factors are defined by the residual variance that is not shared with withdrawal. This allows for precise interpretation of these dimensions, as well as their relationships with external variables (see also Eid et al., 2017, 2018). This approach allows for a more flexible exploration of both the commonalities between dimensions and the specific contributions of each dimension, while having a clear determinant of these commonalities and specific contributions via the chosen reference domain.

An interesting finding is that, although at the first-order level the structure of conflict management was similar between adolescents and adults, at the higher bifactor level, this structure is different. Positive problem solving seemed to be independent from the general factor in adolescence but became part of it in adulthood. In adulthood, positive and negative conflict management strategies were no longer separate dimensions, but as partly unique and partly overlapping dimensions. These findings are supported by the first-order correlations between the dimensions. Positive problem solving did not correlate with the negative dimensions in youths but did so in adults. The correlations between the negative dimensions of conflict engagement, withdrawal and compliance were much higher in youths than in adults. These findings suggest youths differentiate less between withdrawal, compliance and conflict engagement as compared to adults. This means that, as individuals get older and more experienced in managing conflicts and relationships, they have a much clearer inner structure and more flexible and consistent use of the conflict management dimensions. The differences in the structure of the general factor between youths and adults could be explained by the fact that, as children mature, they increase their ability for perspective-taking (Van der Graaff et al., 2014), they diversify their relationships (Smetana et al., 1991; Laursen, 1995; van Doorn et al., 2011a), they learn more sophisticated social skills (Laursen et al., 2001; van Doorn et al., 2011a) and, as a result, they are more capable to adjust their behavioral responses toward others (Laursen et al., 1998; Eichelsheim et al., 2009). Overall, the differences in the communal part of conflict management strategies suggests that the structure of conflict management cannot be considered developmentally homogeneous at the higher-level, which confirms our hypothesis regarding differences between adults and adolescents in the structure of conflict management.

Our validation analyses showed that similar to previous studies (Branje et al., 2009; Bonache et al., 2016), specific dimensions of negative conflict management such as conflict engagement and compliance were associated with negative factors such as higher conflict, negative interactions, and adjustment problems. Positive factors such as perceived tolerance and support and outcomes such as prosocial behaviors were linked to the use of positive problem solving. This dichotomy of negative associated with negative and positive with positive shows that choosing to study only negative or only positive dimensions would limit the ability to understand the effect of conflict management strategies on adjustment. Conversely, the general factor was positively linked to negative factors and outcomes, and negatively linked to positive factors and outcomes. Including a general factor allows understanding whether the commonalities between the dimensions explain the associations with outcomes, and whether the specific dimensions have contributions aside from their commonalities. Our study showed that both the general and the specific dimensions are linked to relationship factors and adjustment outcomes. The proposed bifactorial structure of the construct of conflict management increased the explanatory power when studying associations with psychosocial adjustment. As the general bifactorial structure of conflict management has a higher explanatory power as compared to each individual dimension regarding psychosocial adjustment, it is advisable that, when used for applied purposes such as prevention or intervention, researchers should use factor scores for both general and specific factors, rather than mean scores for individual dimensions.

### 4.1 Limitations and strengths

Our study is not without limitations. First, we used only participants' self-reports on their conflict management strategies with significant others. This might increase desirability, as participants might over report positive problem-solving, while underreporting negative conflict management strategies such as conflict engagement (Laursen et al., 2001). Several studies have shown that, from childhood to adulthood, the discrepancies between observed behavior and self-reports decrease (Laursen et al., 2001). Moreover, self-reports of conflict management strategies have been linked to behavioral outcomes (Branje et al., 2009; van Doorn et al., 2011a) similar to observed conflict management strategies (Laursen et al., 2001), which reinforces the value of such type of assessment. Another limitation stemmed from the complexity of a bifactor model in the validation analyses: we had to split the test for the criterion validity into four different models, as a model with all these variables together did not converge or had unacceptable model fit stemming from penalties for model complexity. Another limitation stems from the fact that we used a normative sample, and the structure of conflict management might be different in samples of individuals with problems. Individuals who exhibit problematic behavior engage more often in conflict and use more negative conflict management strategies such as conflict engagement (e.g., van Doorn et al., 2008). We also allowed several correlated errors in our models. There are some counterindications for this practice: correlated errors might indicate item redundancy and items not being endorsed well by the factor (see Brown, 2015). Nevertheless, all the items for which we allowed correlated errors had good factor loadings on their specific dimensions that were not influenced by the correlated errors, and there are methodologists supporting such practices, especially in complex models such as bifactor models, as these correlated errors account for shared method variance due to the wording of the items (Brown, 2015). For the first wave of youth-reported conflict management, almost half of the youths received a shorter version of the inventory as per research design. This means the results for this first wave should be interpreted with caution. We did find similar results in the rest of waves in which youths reported on their relationships with significant others. We used a significance measure when choosing the best model fit, which is exact and as such arbitrary. Nevertheless, we looked over many models and this structure was validated repeatedly. We were also guided by theory, and we were lenient when model fit was slightly lower than the cut-off value.

Our study has several strengths. First, we have multiple respondents, both adolescents and adults, and two contexts (family and peers), which allows us to explore the structure of conflict management not only across different age groups, but also across different types of relationships, covering both voluntary vs. involuntary and vertical vs. horizontal relationships. This increases the external validity of our measure. Second, we applied an approach of increased complexity culminating in bifactor models, which allowed us to select the structure that fits the best. Third, we used factors from the literature and an extensive longitudinal dataset, which allowed us to test the validity of the structure of conflict management over time. Overall, using different respondents across different relationships, we have not only thoroughly examined the structure of conflict management over adolescence and adulthood, but we have also offered a picture of what the general conflict management factor represents, and how it is linked to adjustment in both adults and adolescents. We have confirmed the existence of the two theoretical axes of positive vs. negative and engagement vs. disengagement via the first order factor analyses which showed these are distinct dimensions. We have enriched the theory on conflict management strategies via showing that the dimensions have commonalities that can be explained by a general factor in which withdrawal is the reference domain.

### 4.2 Implications

Our study suggests the structure of conflict management strategies is more complex than previously presented. First, our study shows that a general factor with withdrawal as reference domain accounts for the commonalities between the dimensions, and also that there are unique contributions of its three specific dimensions, aside from their commonalities accounted by the general factor. Second, a bifactor structure revealed differences in the structure of conflict management between youths and adults that were not captured by first-order factor analyses (Kurdek, 1994, 1995; Bonache et al., 2016). Compared to adults, youths seem to distinguish less between the various negative conflict management strategies, as shown by the high correlations between these dimensions in youths, as well as by the fact that, unlike in adults, the general factor of conflict management did not include the positive problem-solving dimension in youths. While negative conflict management strategies are linked to negative adjustment, not all of these strategies are equally detrimental, especially when the type of relationship is considered (Laursen, 1995). Future studies should explore in-depth the implications of these differences between adults and youths. Third, a bifactor model could solve the discriminant validity issues of conflict management strategies encountered in previous research, especially in adults (Bonache et al., 2016). Previous research found that positive problem-solving was not a good predictor of adjustment problems, while conflict engagement was (e.g., Bonache et al., 2016). We found that positive conflict management associated with positive adjustment, and negative with negative at the level of specific factors. Nevertheless, the general factor of maladaptive conflict management was linked to both positive and negative adjustment. The general factor had better discriminative power than any of the specific dimensions alone. Future research should further explore the link between the general and specific factors of conflict management and adjustment in both youths and adults. Moreover, rather than reducing the use of conflict engagement or increasing the use of positive problem solving as prevention for adjustment problems, interventions could focus on reducing the overall use of negative conflict management strategies.

## Data availability statement

The data analyzed in this study is subject to the following licenses/restrictions: Data is part of an ongoing larger study and any access needs to pass through approval of the project management. Requests to access these datasets should be directed to branje@uu.nl; A.I.Becht@uu.nl.

## Ethics statement

The studies involving humans were approved by the Ethics Review Board of Faculty of Social and Behavioral Sciences at Utrecht University, Netherlands. The studies were conducted in accordance with the local legislation and institutional requirements. Written informed consent for participation in this study was provided by the participants' legal guardians/next of kin.

## Author contributions

TT and SB participated in the design and conception of the current study. TT drafted the manuscript and performed the statistical analyses. SB and WM designed the multiple-respondent project. WM offered comments on the manuscript. All authors contributed to the article and approved the submitted version.
