# Supplementary material for: The dimensionality of the Conflict Resolution Styles Inventory across age and relationships
Source: Front Psychol. 2024 Mar 12;15:1233279. doi: 10.3389/fpsyg.2024.1233279 (PMC10963530; doi:10.3389/fpsyg.2024.1233279)
Supplement: Supplementary file 1 [file Data_Sheet_1.docx]

Supplementary Material

The Dimensionality of Conflict Resolution Styles Inventory Across Age and Relationships

Tatiana Alina Trifan*, Wim Meeus and Susan Branje

*** Correspondence:** Corresponding Author: [tatiana.trifan@gmail.com](mailto:tatiana.trifan@gmail.com); [t.a.trifan@uu.nl](mailto:t.a.trifan@uu.nl)

## Supplementary Tables

**Table 1**

*Model fit indices for Confirmatory Factor Analysis models for each type of respondent*

| Adolescent-Best friend | | | | | | | |
| --- | --- | --- | --- | --- | --- | --- | --- |
|  | *X*^2^ | *df* | Sig. | CFI/TLI | RMSEA [90% C.I.] | RMSEA ≤ .05 | SRMR |
| Wave 1 | 339.041 | 161 | .000 | 0.93/0.92 | 0.049 [0.042 - 0.056] | 0.595 | 0.058 |
| Wave 2 | 258.430 | 84 | .000 | 0.93/0.91 | 0.068 [0.059 - 0.078] | 0.001 | 0.057 |
| Wave 3 | 187.294 | 85 | .000 | 0.96/0.95 | 0.053 [0.043 - 0.063] | 0.314 | 0.049 |
| Wave 4 | 355.024 | 161 | .000 | 0.95/0.94 | 0.054 [0.046 - 0.061] | 0.215 | 0.045 |
| Wave 5 | 402.643 | 161 | .000 | 0.94/0.93 | 0.061 [0.054 - 0.069] | 0.007 | 0.045 |
| Wave 6 | 387.568 | 161 | .000 | 0.95/0.94 | 0.060 [0.053 - 0.068] | 0.015 | 0.041 |
| Best friend-Adolescent | | | | | | | |
|  | *X*^2^ | *df* | Sig. | CFI/TLI | RMSEA [90% C.I.] | RMSEA ≤ .05 | SRMR |
| Wave 1 | 278.770 | 161 | .000 | 0.93/0.92 | 0.040 [0.032 - 0.048] | 0.978 | 0.057 |
| Wave 2 | 185.575 | 85 | .000 | 0.93/0.91 | 0.053 [0.043 - 0.063] | 0.301 | 0.053 |
| Wave 3 | 210.025 | 85 | .000 | 0.93/0.92 | 0.059 [0.049 - 0.069] | 0.064 | 0.056 |
| Wave 4 | 363.703 | 161 | .000 | 0.93/0.92 | 0.055 [0.048 - 0.063] | 0.125 | 0.057 |
| Wave 5 | 385.746 | 161 | .000 | 0.93/0.91 | 0.060 [0.052 - 0.067] | 0.020 | 0.057 |
| Wave 6 | 328.133 | 161 | .000 | 0.94/0.93 | 0.053 [0.045 - 0.061] | 0.259 | 0.053 |
| Adolescent-Mother | | | | | | | |
|  | *X*^2^ | *df* | Sig. | CFI/TLI | RMSEA [90% C.I.] | RMSEA ≤ .05 | SRMR |
| Wave 1 | 312.967 | 161 | .000 | 0.95/0.94 | 0.044 [0.036 - 0.051] | 0.922 | 0.055 |
| Wave 2 | 146.350 | 85 | .000 | 0.98/0.97 | 0.039 [0.028 - 0.050] | 0.950 | 0.042 |
| Wave 3 | 150.800 | 85 | .000 | 0.97/0.97 | 0.042 [0.031 - 0.052] | 0.900 | 0.038 |
| Wave 4 | 403.610 | 161 | .000 | 0.94/0.93 | 0.059 [0.052 - 0.066] | 0.023 | 0.052 |
| Wave 5 | 363.253 | 161 | .000 | 0.95/0.94 | 0.055 [0.047 - 0.062] | 0.147 | 0.050 |
| Wave 6 | 370.973 | 161 | .000 | 0.95/0.94 | 0.056 [0.048 - 0.063] | 0.107 | 0.050 |
| Adolescent-Father | | | | | | | |
|  | *X*^2^ | *df* | Sig. | CFI/TLI | RMSEA [90% C.I.] | RMSEA ≤ .05 | SRMR |
| Wave 1 | 319.532 | 161 | .000 | 0.93/0.91 | 0.046 [0.039 - 0.053] | 0.804 | 0.062 |
| Wave 2 | 170.330 | 85 | .000 | 0.96/0.95 | 0.047 [0.037 - 0.057] | 0.661 | 0.059 |
| Wave 3 | 167.071 | 85 | .000 | 0.96/0.95 | 0.047 [0.037 - 0.058] | 0.658 | 0.043 |
| Wave 4 | 423.180 | 161 | .000 | 0.91/0.90 | 0.062 [0.055 -0.070] | 0.003 | 0.066 |
| Wave 5 | 419.264 | 161 | .000 | 0.92/0.91 | 0.063 [0.056 - 0.071] | 0.002 | 0.063 |
| Wave 6 | 442.302 | 161 | .000 | 0.93/0.91 | 0.066 [0.058 - 0.073] | 0.000 | 0.064 |
| Sibling-Mother | | | | | | | |
|  | *X*^2^ | *df* | Sig. | CFI/TLI | RMSEA [90% C.I.] | RMSEA ≤ .05 | SRMR |
| Wave 1 | 323.099 | 161 | .000 | 0.92/0.90 | 0.049 [0.041 - 0.057] | 0.582 | 0.064 |
| Wave 2 | 226.063 | 85 | .000 | 0.93/0.91 | 0.064 [0.054 - 0.075] | 0.010 | 0.063 |
| Wave 3 | 222.616 | 85 | .000 | 0.93/0.92 | 0.064 [0.054 - 0.075] | 0.011 | 0.059 |
| Wave 4 | 413.380 | 161 | .000 | 0.92/0.90 | 0.064 [0.056 - 0.072] | 0.001 | 0.064 |
| Wave 5 | 445.167 | 161 | .000 | 0.91/0.89 | 0.070 [0.062 - 0.077] | 0.000 | 0.059 |
| Wave 6 | 354.334 | 161 | .000 | 0.94/0.93 | 0.058 [0.050 - 0.066] | 0.056 | 0.054 |
| Mother-Adolescent | | | | | | | |
|  | *X*^2^ | *df* | Sig. | CFI/TLI | RMSEA [90% C.I.] | RMSEA ≤ .05 | SRMR |
| Wave 1 | 400.147 | 161 | .000 | 0.91/0.90 | 0.055 [0.048 - 0.062] | 0.118 | 0.054 |
| Wave 2 | 280.035 | 161 | .000 | 0.94/0.93 | 0.040 [0.032 - 0.048] | 0.984 | 0.043 |
| Wave 3 | 376.796 | 161 | .000 | 0.93/0.91 | 0.054 [0.047 - 0.061] | 0.154 | 0.060 |
| Wave 4 | 430.323 | 161 | .000 | 0.91/0.90 | 0.062 [0.055 - 0.069] | 0.003 | 0.060 |
| Wave 5 | 283.873 | 161 | .000 | 0.96/0.95 | 0.043 [0.034 - 0.051] | 0.931 | 0.050 |
| Wave 6 | 390.174 | 161 | .000 | 0.93/0.91 | 0.058 [0.051 - 0.066] | 0.032 | 0.052 |
| Father-Adolescent | | | | | | | |
|  | *X*^2^ | *df* | Sig. | CFI/TLI | RMSEA [90% C.I.] | RMSEA ≤ .05 | SRMR |
| Wave 1 | 340.034 | 161 | .000 | 0.93/0.91 | 0.050 [0.043 - 0.057] | 0.490 | 0.056 |
| Wave 2 | 427.807 | 161 | .000 | 0.90/0.88 | 0.063 [0.055 - 0.070] | 0.002 | 0.066 |
| Wave 3 | 402.114 | 161 | .000 | 0.91/0.89 | 0.060 [0.053 - 0.068] | 0.012 | 0.059 |
| Wave 4 | 370.932 | 161 | .000 | 0.92/0.90 | 0.057 [0.050 - 0.065] | 0.061 | 0.062 |
| Wave 5 | 384.746 | 161 | .000 | 0.92/0.90 | 0.061 [0.053 - 0.069] | 0.012 | 0.062 |
| Wave 6 | 407.158 | 161 | .000 | 0.92/0.90 | 0.064 [0.056 - 0.072] | 0.002 | 0.059 |
| Mother-Father | | | | | | | |
|  | *X*^2^ | *df* | Sig. | CFI/TLI | RMSEA [90% C.I.] | RMSEA ≤ .05 | SRMR |
| Wave 1 | 366.555 | 161 | .000 | 0.94/0.93 | 0.053 [0.046 - 0.060] | 0.225 | 0.050 |
| Wave 2 | 527.349 | 161 | .000 | 0.90/0.88 | 0.073 [0.066 - 0.080] | 0.000 | 0.064 |
| Wave 3 | 470.729 | 161 | .000 | 0.92/0.90 | 0.068 [0.061 - 0.075] | 0.000 | 0.058 |
| Wave 4 | 341.325 | 161 | .000 | 0.95/0.94 | 0.052 [0.045 - 0.060] | 0.296 | 0.047 |
| Wave 5 | 358.742 | 161 | .000 | 0.94/0.93 | 0.057 [0.049 - 0.065] | 0.068 | 0.050 |
| Wave 6 | 399.792 | 161 | .000 | 0.94/0.93 | 0.063 [0.055 - 0.071] | 0.004 | 0.054 |
| Father-Mother | | | | | | | |
|  | *X*^2^ | *df* | Sig. | CFI/TLI | RMSEA [90% C.I.] | RMSEA ≤ .05 | SRMR |
| Wave 1 | 420.347 | 161 | .000 | 0.93/0.92 | 0.060 [0.053 - 0.067] | 0.008 | 0.054 |
| Wave 2 | 393.667 | 161 | .000 | 0.93/0.92 | 0.059 [0.051 - 0.066] | 0.025 | 0.057 |
| Wave 3 | 422.490 | 161 | .000 | 0.93/0.92 | 0.063 [0.056 - 0.070] | 0.002 | 0.051 |
| Wave 4 | 411.280 | 161 | .000 | 0.93/0.92 | 0.063 [0.055 - 0.070] | 0.003 | 0.057 |
| Wave 5 | 356.284 | 161 | .000 | 0.95/0.94 | 0.057 [0.049 - 0.065] | 0.064 | 0.048 |
| Wave 6 | 308.812 | 161 | .000 | 0.96/0.95 | 0.050 [0.042 - 0.059] | 0.471 | 0.046 |
| Adolescent-Intimate partner | | | | | | | |
|  | *X*^2^ | *df* | Sig. | CFI/TLI | RMSEA [90% C.I.] | RMSEA ≤ .05 | SRMR |
| Wave 7 | 289.566 | 161 | .000 | 0.91/0.89 | 0.068 [0.056 - 0.081] | 0.011 | 0.070 |
| Wave 8 | 236.531 | 161 | .000 | 0.95/0.94 | 0.051 [0.036 - 0.064] | 0.457 | 0.059 |
| Wave 9 | 281.623 | 161 | .000 | 0.93/0.92 | 0.059 [0.048 - 0.071] | 0.092 | 0.061 |
| Intimate partner-Adolescent | | | | | | | |
|  | *X*^2^ | *df* | Sig. | CFI/TLI | RMSEA [90% C.I.] | RMSEA ≤ .05 | SRMR |
| Wave 7 | 232.166 | 161 | .000 | 0.93/0.92 | 0.054 [0.038 - 0.069] | 0.321 | 0.077 |
| Wave 8 | 216.046 | 161 | .000 | 0.95/0.94 | 0.046 [0.028 - 0.062] | 0.640 | 0.067 |
| Wave 9 | 248.299 | 161 | .000 | 0.93/0.92 | 0.055 [0.041 - 0.068] | 0.267 | 0.068 |

*Note.* For these models, we used Maximum Likelihood (ML) as estimator.

X^2^ = Chi-square, *df* = degrees of freedom, CFI = comparative fit index, TLI = Tucker-Lewis index, RMSEA = root mean square error of approximation, SRMR = standardized root mean square residual.

**Table 2**

*Model fit indices for CFA-ESEM models for each respondent*

| Adolescent-Best friend | | | | | | | |
| --- | --- | --- | --- | --- | --- | --- | --- |
|  | *X*^2^ | *df* | Sig. | CFI/TLI | RMSEA [90% C.I.] | RMSEA ≤ .05 | SRMR |
| Wave 1 | 195.814 | 113 | .000 | 0.97/0.95 | 0.040 [0.030 - 0.049] | 0.967 | 0.034 |
| Wave 2 | 168.255 | 61 | .000 | 0.96/0.92 | 0.063 [0.051 - 0.074] | 0.032 | 0.027 |
| Wave 3 | 121.864 | 61 | .000 | 0.97/0.95 | 0.048 [0.036 - 0.060] | 0.584 | 0.023 |
| Wave 4 | 265.417 | 113 | .000 | 0.96/0.93 | 0.057 [0.048 - 0.065] | 0.106 | 0.026 |
| Wave 5 | 297.621 | 113 | .000 | 0.95/0.92 | 0.064 [0.055 - 0.073] | 0.005 | 0.029 |
| Wave 6 | 284.640 | 113 | .000 | 0.96/0.94 | 0.062 [0.053 - 0.072] | 0.012 | 0.024 |
| Best friend-Adolescent | | | | | | | |
|  | *X*^2^ | *df* | Sig. | CFI/TLI | RMSEA [90% C.I.] | RMSEA ≤ .05 | SRMR |
| Wave 1 | 160.634 | 113 | .002 | 0.97/0.95 | 0.031 [0.019 - 0.041] | 0.999 | 0.031 |
| Wave 2 | 115.076 | 61 | .000 | 0.96/0.94 | 0.046 [0.033 - 0.059] | 0.684 | 0.027 |
| Wave 3 | 124.568 | 61 | .000 | 0.97/0.94 | 0.050 [0.037 - 0.062] | 0.488 | 0.026 |
| Wave 4 | 216.558 | 113 | .000 | 0.97/0.94 | 0.047 [0.038 - 0.057] | 0.682 | 0.028 |
| Wave 5 | 208.622 | 113 | .000 | 0.97/0.95 | 0.046 [0.036 - 0.056] | 0.720 | 0.027 |
| Wave 6 | 211.464 | 113 | .000 | 0.96/0.94 | 0.049 [0.038 - 0.059] | 0.573 | 0.028 |
| Adolescent-Mother | | | | | | | |
|  | *X*^2^ | *df* | Sig. | CFI/TLI | RMSEA [90% C.I.] | RMSEA ≤ .05 | SRMR |
| Wave 1 | 173.843 | 113 | .000 | 0.98/0.96 | 0.033 [0.023 - 0.042] | 0.999 | 0.029 |
| Wave 2 | 101.360 | 61 | .001 | 0.98/0.97 | 0.038 [0.024 - 0.050] | 0.944 | 0.023 |
| Wave 3 | 133.600 | 61 | .000 | 0.97/0.95 | 0.052 [0.040 - 0.063] | 0.398 | 0.028 |
| Wave 4 | 224.366 | 113 | .000 | 0.97/0.95 | 0.047 [0.038 - 0.057] | 0.664 | 0.022 |
| Wave 5 | 225.748 | 113 | .000 | 0.97/0.95 | 0.049 [0.039 - 0.058] | 0.576 | 0.024 |
| Wave 6 | 211.836 | 113 | .000 | 0.98/0.96 | 0.045 [0.036 - 0.055] | 0.778 | 0.020 |
| Adolescent-Father | | | | | | | |
|  | *X*^2^ | *df* | Sig. | CFI/TLI | RMSEA [90% C.I.] | RMSEA ≤ .05 | SRMR |
| Wave 1 | 188.196 | 113 | .000 | 0.96/0.94 | 0.038 [0.028 - 0.047] | 0.985 | 0.030 |
| Wave 2 | 72.201 | 61 | .154 | 0.99/0.99 | 0.020 [0.000 - 0.036] | 1.000 | 0.020 |
| Wave 3 | 133.820 | 61 | .000 | 0.96/0.94 | 0.052 [0.040 - 0.065] | 0.353 | 0.029 |
| Wave 4 | 211.112 | 113 | .000 | 0.97/0.95 | 0.046 [0.036 - 0.055] | 0.771 | 0.027 |
| Wave 5 | 216.656 | 113 | .000 | 0.97/0.95 | 0.048 [0.038 - 0.057] | 0.637 | 0.025 |
| Wave 6 | 211.924 | 113 | .000 | 0.97/0.96 | 0.046 [0.037 - 0.056] | 0.716 | 0.022 |
| Sibling-Mother | | | | | | | |
|  | *X*^2^ | *df* | Sig. | CFI/TLI | RMSEA [90% C.I.] | RMSEA ≤ .05 | SRMR |
| Wave 1 | 181.471 | 113 | .000 | 0.96/0.94 | 0.038 [0.027 - 0.048] | 0.978 | 0.036 |
| Wave 2 | 126.489 | 61 | .000 | 0.97/0.94 | 0.052 [0.039 - 0.065] | 0.390 | 0.030 |
| Wave 3 | 145.744 | 61 | .000 | 0.96/0.93 | 0.060 [0.047 - 0.072] | 0.096 | 0.030 |
| Wave 4 | 214.636 | 113 | .000 | 0.97/0.94 | 0.049 [0.039 - 0.058] | 0.584 | 0.029 |
| Wave 5 | 257.171 | 113 | .000 | 0.95/0.92 | 0.059 [0.050 - 0.069] | 0.058 | 0.029 |
| Wave 6 | 229.099 | 113 | .000 | 0.96/0.94 | 0.053 [0.043 - 0.063] | 0.272 | 0.026 |
| Mother-Adolescent | | | | | | | |
|  | *X*^2^ | *df* | Sig. | CFI/TLI | RMSEA [90% C.I.] | RMSEA ≤ .05 | SRMR |
| Wave 1 | 173.843 | 113 | .000 | 0.98/0.96 | 0.033 [0.023 - 0.042] | 0.999 | 0.029 |
| Wave 2 | 168.225 | 113 | .000 | 0.98/0.96 | 0.033 [0.022 - 0.042] | 0.999 | 0.025 |
| Wave 3 | 182.965 | 113 | .000 | 0.98/0.96 | 0.037 [0.027 - 0.047] | 0.989 | 0.025 |
| Wave 4 | 226.057 | 113 | .000 | 0.96/0.94 | 0.048 [0.039 - 0.057] | 0.648 | 0.027 |
| Wave 5 | 174.861 | 113 | .000 | 0.98/0.96 | 0.036 [0.025 - 0.046] | 0.989 | 0.024 |
| Wave 6 | 246.779 | 113 | .000 | 0.96/0.93 | 0.053 [0.044 - 0.062] | 0.273 | 0.028 |
| Father-Adolescent | | | | | | | |
|  | *X*^2^ | *df* | Sig. | CFI/TLI | RMSEA [90% C.I.] | RMSEA ≤ .05 | SRMR |
| Wave 1 | 190.358 | 113 | .000 | 0.97/0.94 | 0.039 [0.029 - 0.049] | 0.970 | 0.029 |
| Wave 2 | 220.416 | 113 | .000 | 0.96/0.93 | 0.047 [0.038 - 0.057] | 0.670 | 0.028 |
| Wave 3 | 216.972 | 113 | .000 | 0.96/0.93 | 0.047 [0.038 - 0.057] | 0.680 | 0.029 |
| Wave 4 | 196.940 | 113 | .000 | 0.97/0.95 | 0.043 [0.033 - 0.053] | 0.868 | 0.026 |
| Wave 5 | 226.451 | 113 | .000 | 0.96/0.93 | 0.052 [0.042 - 0.062] | 0.372 | 0.027 |
| Wave 6 | 241.974 | 113 | .000 | 0.96/0.93 | 0.055 [0.046 - 0.065] | 0.175 | 0.028 |
| Mother-Father | | | | | | | |
|  | *X*^2^ | *df* | Sig. | CFI/TLI | RMSEA [90% C.I.] | RMSEA ≤ .05 | SRMR |
| Wave 1 | 204.154 | 113 | .000 | 0.97/0.96 | 0.042 [0.033 - 0.051] | 0.914 | 0.023 |
| Wave 2 | 273.963 | 113 | .000 | 0.96/0.92 | 0.058 [0.049 - 0.066] | 0.074 | 0.026 |
| Wave 3 | 268.426 | 113 | .000 | 0.96/0.93 | 0.057 [0.048 - 0.066] | 0.085 | 0.025 |
| Wave 4 | 216.070 | 113 | .000 | 0.97/0.95 | 0.047 [0.038 - 0.057] | 0.669 | 0.024 |
| Wave 5 | 227.414 | 113 | .000 | 0.97/0.95 | 0.052 [0.042 - 0.062] | 0.362 | 0.024 |
| Wave 6 | 233.518 | 113 | .000 | 0.97/0.95 | 0.053 [0.044 - 0.063] | 0.280 | 0.023 |
| Father-Mother | | | | | | | |
|  | *X*^2^ | *df* | Sig. | CFI/TLI | RMSEA [90% C.I.] | RMSEA ≤ .05 | SRMR |
| Wave 1 | 234.649 | 113 | .000 | 0.97/0.95 | 0.049 [0.040 - 0.058] | 0.534 | 0.025 |
| Wave 2 | 188.829 | 113 | .000 | 0.98/0.96 | 0.040 [0.30 - 0.050] | 0.952 | 0.021 |
| Wave 3 | 237.372 | 113 | .000 | 0.97/0.95 | 0.052 [0.043 - 0.061] | 0.353 | 0.022 |
| Wave 4 | 200.474 | 113 | .000 | 0.98/0.96 | 0.044 [0.034 - 0.054] | 0.817 | 0.022 |
| Wave 5 | 230.549 | 113 | .000 | 0.97/0.95 | 0.053 [0.043 - 0.063] | 0.217 | 0.023 |
| Wave 6 | 178.948 | 113 | .000 | 0.98/0.97 | 0.040 [0.029 - 0.051] | 0.934 | 0.021 |
| Adolescent-Intimate partner | | | | | | | |
|  | *X*^2^ | *df* | Sig. | CFI/TLI | RMSEA [90% C.I.] | RMSEA ≤ .05 | SRMR |
| Wave 7 | 186.072 | 113 | .000 | 0.95/0.91 | 0.061 [0.045 - 0.077] | 0.116 | 0.034 |
| Wave 8 | 141.456 | 113 | .000 | 0.98/0.97 | 0.037 [0.010 - 0.055] | 0.871 | 0.029 |
| Wave 9 | 190.063 | 113 | .000 | 0.95/0.92 | 0.057 [0.042 - 0.070] | 0.213 | 0.033 |
| Intimate partner-Adolescent | | | | | | | |
|  | *X*^2^ | *df* | Sig. | CFI/TLI | RMSEA [90% C.I.] | RMSEA ≤ .05 | SRMR |
| Wave 7 | 133.545 | 113 | .091 | 0.98/0.97 | 0.035 [0.000 - 0.056] | 0.869 | 0.033 |
| Wave 8 | 124.534 | 113 | .215 | 0.99/0.98 | 0.025 [0.000 - 0.049] | 0.960 | 0.031 |
| Wave 9 | 131.072 | 113 | .117 | 0.99/0.98 | 0.030 [0.000 - 0.050] | 0.951 | 0.029 |

*Note.* For these models, we used Maximum Likelihood (ML) as estimator.

X^2^ = Chi-square, *df* = degrees of freedom, CFI = comparative fit index, TLI = Tucker-Lewis index, RMSEA = root mean square error of approximation, SRMR = standardized root mean square residual.

**Table 3**

*Model fit indices for Confirmatory Factor Analysis models for grouped respondents with nestedness accounted*

| Adolescent-Best friend/ Best friend-Adolescent (N1=911, N2=868, N3=851, N4=834, N5=795, N6=757) | | | | | | | |
| --- | --- | --- | --- | --- | --- | --- | --- |
|  | *X*^2^ | *df* | Sig. | CFI/TLI | R RMSEA [90% C.I.] | RMSEA ≤ .05 | SRMR |
| Wave 1 | 354.811 | 161 | .000 | 0.94/0.93 | 0.036 [0.031 - 0.041] | 1.000 | 0.051 |
| Wave 2 | 252.856 | 85 | .000 | 0.94/0.93 | 0.048 [0.041 - 0.055] | 0.702 | 0.050 |
| Wave 3 | 245.056 | 85 | .000 | 0.95/0.93 | 0.047 [0.040 - 0.054] | 0.751 | 0.048 |
| Wave 4 | 545.030 | 161 | .000 | 0.92/0.91 | 0.053 [0.049 - 0.058] | 0.120 | 0.050 |
| Wave 5 | 488.236 | 161 | .000 | 0.93/0.92 | 0.051 [0.045 - 0.056] | 0.419 | 0.047 |
| Wave 6 | 369.379 | 161 | .000 | 0.96/0.95 | 0.041 [0.036 - 0.047] | 0.995 | 0.042 |
| Adolescent-Mother/ Adolescent-Father/ Sibling-Mother (N1=1378, N2=1316, N3=1272, N4=1237, N5=1187, N6=1187) | | | | | | | |
|  | *X*^2^ | *df* | Sig. | CFI/TLI | RMSEA [90% C.I.] | RMSEA ≤ .05 | SRMR |
| Wave 1 | 529.859 | 161 | .000 | 0.93/0.92 | 0.041 [0.037 - 0.045] | 1.000 | 0.054 |
| Wave 2 | 238.083 | 85 | .000 | 0.97/0.96 | 0.037 [0.031 - 0.043] | 1.000 | 0.042 |
| Wave 3 | 260.895 | 85 | .000 | 0.96/0.95 | 0.040 [0.035 - 0.046] | 0.998 | 0.037 |
| Wave 4 | 645.066 | 161 | .000 | 0.93/0.92 | 0.049 [0.045 - 0.053] | 0.606 | 0.053 |
| Wave 5 | 652.316 | 161 | .000 | 0.93/0.92 | 0.051 [0.047 - 0.055] | 0.380 | 0.051 |
| Wave 6 | 586.849 | 161 | .000 | 0.95/0.94 | 0.047 [0.043 - 0.051] | 0.865 | 0.050 |
| Mother-Adolescent/ Father-Adolescent (N1=940, N2=885, N3=868, N4=838, N5=794, N6=792) | | | | | | | |
|  | *X*^2^ | *df* | Sig. | CFI/TLI | RMSEA [90% C.I.] | RMSEA ≤ .05 | SRMR |
| Wave 1 | 486.721 | 161 | .000 | 0.92/0.91 | 0.046 [0.042 - 0.051] | 0.893 | 0.049 |
| Wave 2 | 507.394 | 161 | .000 | 0.92/0.91 | 0.049 [0.045 - 0.054] | 0.585 | 0.052 |
| Wave 3 | 517.615 | 161 | .000 | 0.92/0.91 | 0.051 [0.046 - 0.055] | 0.422 | 0.054 |
| Wave 4 | 531.476 | 161 | .000 | 0.92/0.90 | 0.052 [0.047 - 0.057] | 0.206 | 0.056 |
| Wave 5 | 429.244 | 161 | .000 | 0.94/0.93 | 0.046 [0.041 - 0.051] | 0.903 | 0.051 |
| Wave 6 | 550.964 | 161 | .000 | 0.92/0.91 | 0.055 [0.050 - 0.060] | 0.041 | 0.051 |
| Mother-Father/ Father-Mother (N1=893, N2=848, N3=827, N4=801, N5=744, N6=740) | | | | | | | |
|  | *X*^2^ | *df* | Sig. | CFI/TLI | RMSEA [90% C.I.] | RMSEA ≤ .05 | SRMR |
| Wave 1 | 562.302 | 161 | .000 | 0.94/0.93 | 0.053 [0.048 - 0.058] | 0.159 | 0.050 |
| Wave 2 | 656.876 | 161 | .000 | 0.91/0.90 | 0.060 [0.056 - 0.065] | 0.000 | 0.058 |
| Wave 3 | 628.303 | 161 | .000 | 0.92/0.91 | 0.059 [0.054 - 0.064] | 0.001 | 0.054 |
| Wave 4 | 560.940 | 161 | .000 | 0.93/0.91 | 0.056 [0.051 - 0.061] | 0.030 | 0.051 |
| Wave 5 | 514.951 | 161 | .000 | 0.94/0.93 | 0.054 [0.049 - 0.060] | 0.084 | 0.047 |
| Wave 6 | 474.750 | 161 | .000 | 0.95/0.94 | 0.051 [0.046 - 0.057] | 0.334 | 0.046 |
| Adolescent-Intimate partner/ Intimate partner-Adolescent (N7=322, N8= 343, N9=393) | | | | | | | |
|  | *X*^2^ | *df* | Sig. | CFI/TLI | RMSEA [90% C.I.] | RMSEA ≤ .05 | SRMR |
| Wave 7 | 273.219 | 161 | .000 | 0.94/0.93 | 0.047 [0.037 - 0.056] | 0.719 | 0.060 |
| Wave 8 | 246.256 | 161 | .000 | 0.96/0.95 | 0.039 [0.029 - 0.049] | 0.969 | 0.052 |
| Wave 9 | 327.144 | 161 | .000 | 0.93/0.92 | 0.051 [0.043 - 0.059] | 0.388 | 0.053 |

*Note.* We used Maximum Likelihood Robust (MLR) as estimator, due to nestedness.

X^2^ = Chi-square, *df* = degrees of freedom, CFI = comparative fit index, TLI = Tucker-Lewis index, RMSEA = root mean square error of approximation, SRMR = standardized root mean square residual.

**Table 4**

*Model fit indices for second-order factor models for grouped respondents with nestedness accounted*

| Positivity - Adolescent-Best friend/ Best friend-Adolescent (N1=911, N2=868, N3=851, N4=834, N5=795, N6=757) | | | | | | | |
| --- | --- | --- | --- | --- | --- | --- | --- |
|  | *X*^2^ | *df* | Sig. | CFI/TLI | RMSEA [90% C.I.] | RMSEA ≤ .05 | SRMR |
| Wave 1 | 448.514 | 166 | .000 | 0.92/0.90 | 0.043 [0.038 - 0.048] | 0.990 | 0.071 |
| Wave 4 | 652.967 | 166 | .000 | 0.90/0.89 | 0.059 [0.055 - 0.064] | 0.001 | 0.071 |
| Wave 5 | 547.182 | 166 | .000 | 0.91/0.90 | 0.054 [0.049 - 0.059] | 0.106 | 0.062 |
| Wave 6 | 422.360 | 166 | .000 | 0.95/0.94 | 0.045 [0.040 - 0.051] | 0.930 | 0.055 |
| Engagement - Adolescent-Best friend/ Best friend-Adolescent (N1=911, N2=868, N3=851, N4=834, N5=795, N6=757) | | | | | | | |
|  | *X*^2^ | *df* | Sig. | CFI/TLI | RMSEA [90% C.I.] | RMSEA ≤ .05 | SRMR |
| Wave 1 | 660.596 | 165 | .000 | 0.85/0.83 | 0.057 [0.053 - 0.062] | 0.004 | 0.115 |
| Wave 4 | 839.596 | 165 | .000 | 0.87/0.85 | 0.070 [0.065 - 0.075] | 0.000 | 0.121 |
| Wave 5 | 764.928 | 165 | .000 | 0.88/0.86 | 0.068 [0.063 - 0.073] | 0.000 | 0.130 |
| Wave 6 | 709.792 | 165 | .000 | 0.89/0.87 | 0.066 [0.061 - 0.071] | 0.000 | 0.156 |
| Positivity - Adolescent-Mother/ Adolescent-Father/ Sibling-Mother (N1=1378, N2=1316, N3=1272, N4=1237, N5=1187, N6=1187) | | | | | | | |
|  | *X*^2^ | *df* | Sig. | CFI/TLI | RMSEA [90% C.I.] | RMSEA ≤ .05 | SRMR |
| Wave 1 | 725.439 | 166 | .000 | 0.89/0.88 | 0.049 [0.046 - 0.053] | 0.589 | 0.094 |
| Wave 4 | 818.568 | 166 | .000 | 0.91/0.90 | 0.056 [0.053 - 0.060] | 0.003 | 0.097 |
| Wave 5 | 838.823 | 166 | .000 | 0.91/0.90 | 0.058 [0.055 - 0.062] | 0.000 | 0.097 |
| Wave 6 | 712.365 | 166 | .000 | 0.93/0.92 | 0.053 [0.049 - 0.057] | 0.132 | 0.085 |
| Engagement - Adolescent-Mother/ Adolescent-Father/ Sibling-Mother (N1=1378, N2=1316, N3=1272, N4=1237, N5=1187, N6=1187) | | | | | | | |
|  | *X*^2^ | *df* | Sig. | CFI/TLI | RMSEA [90% C.I.] | RMSEA ≤ .05 | SRMR |
| Wave 1 | 870.364 | 165 | .000 | 0.87/0.85 | 0.056 [0.052 - 0.059] | 0.005 | 0.114 |
| Wave 4 | 968.342 | 165 | .000 | 0.89/0.87 | 0.063 [0.059 - 0.067] | 0.000 | 0.124 |
| Wave 5 | 904.299 | 165 | .000 | 0.90/0.88 | 0.061 [0.058 - 0.065] | 0.000 | 0.114 |
| Wave 6 | 824.835 | 165 | .000 | 0.92/0.91 | 0.058 [0.054 - 0.062] | 0.000 | 0.119 |
| Positivity - Mother-Adolescent/ Father-Adolescent (N1=940, N2=885, N3=868, N4=838, N5=794, N6=792) | | | | | | | |
|  | *X*^2^ | *df* | Sig. | CFI/TLI | RMSEA [90% C.I.] | RMSEA ≤ .05 | SRMR |
| Wave 1 | 622.738 | 166 | .000 | 0.89/0.88 | 0.054 [0.050 - 0.059] | 0.066 | 0.093 |
| Wave 2 | 630.678 | 166 | .000 | 0.90/0.88 | 0.056 [0.052 - 0.061] | 0.014 | 0.096 |
| Wave 3 | 667.948 | 166 | .000 | 0.89/0.87 | 0.059 [0.054 - 0.064] | 0.001 | 0.110 |
| Wave 4 | 664.694 | 166 | .000 | 0.89/0.87 | 0.060 [0.055 - 0.065] | 0.000 | 0.105 |
| Wave 5 | 536.492 | 166 | .000 | 0.92/0.91 | 0.053 [0.048 - 0.058] | 0.156 | 0.107 |
| Wave 6 | 673.947 | 166 | .000 | 0.90/0.88 | 0.052 [0.057 - 0.067] | 0.000 | 0.113 |
| Engagement - Mother-Adolescent/ Father-Adolescent (N1=940, N2=885, N3=868, N4=838, N5=794, N6=792) | | | | | | | |
|  | *X*^2^ | *df* | Sig. | CFI/TLI | RMSEA [90% C.I.] | RMSEA ≤ .05 | SRMR |
| Wave 1 | 699.148 | 165 | .000 | 0.88/0.86 | 0.059 [0.054 - 0.063] | 0.001 | 0.109 |
| Wave 2 | 736.697 | 165 | .000 | 0.87/0.85 | 0.063 [0.058 - 0.067] | 0.000 | 0.117 |
| Wave 3 | 783.279 | 165 | .000 | 0.86/0.84 | 0.066 [0.061 - 0.070] | 0.000 | 0.134 |
| Wave 4 | 728.695 | 165 | .000 | 0.87/0.85 | 0.064 [0.059 - 0.069] | 0.000 | 0.126 |
| Wave 5 | 669.173 | 165 | .000 | 0.89/0.87 | 0.062 [0.057 - 0.067] | 0.000 | 0.139 |
| Wave 6 | 792.439 | 165 | .000 | 0.87/0.85 | 0.069 [0.064 - 0.074] | 0.000 | 0.143 |
| Positivity - Mother-Father/ Father-Mother (N1=893, N2=848, N3=827, N4=801, N5=744, N6=740) | | | | | | | |
|  | *X*^2^ | *df* | Sig. | CFI/TLI | RMSEA [90% C.I.] | RMSEA ≤ .05 | SRMR |
| Wave 1 | 927.600 | 166 | .000 | 0.88/0.86 | 0.072 [0.067 - 0.076] | 0.000 | 0.149 |
| Wave 2 | 956.953 | 166 | .000 | 0.86/0.84 | 0.070 [0.070 - 0.080] | 0.000 | 0.150 |
| Wave 3 | 904.859 | 166 | .000 | 0.88/0.86 | 0.073 [0.069 - 0.078] | 0.000 | 0.148 |
| Wave 4 | 836.180 | 166 | .000 | 0.88/0.86 | 0.071 [0.066 - 0.076] | 0.000 | 0.142 |
| Wave 5 | 755.909 | 166 | .000 | 0.89/0.88 | 0.069 [0.064 - 0.074] | 0.000 | 0.146 |
| Wave 6 | 709.404 | 166 | .000 | 0.91/0.89 | 0.067 [0.062 - 0.072] | 0.000 | 0.153 |
| Engagement - Mother-Father/ Father-Mother (N1=893, N2=848, N3=827, N4=801, N5=744, N6=740) | | | | | | | |
|  | *X*^2^ | *df* | Sig. | CFI/TLI | RMSEA [90% C.I.] | RMSEA ≤ .05 | SRMR |
| Wave 1 | 899.868 | 165 | .000 | 0.88/0.87 | 0.071 [0.066 - 0.075] | 0.000 | 0.152 |
| Wave 2 | 922.733 | 165 | .000 | 0.86/0.84 | 0.074 [0.069 - 0.078] | 0.000 | 0.149 |
| Wave 3 | 900.798 | 165 | .000 | 0.88/0.86 | 0.073 [0.069 - 0.078] | 0.000 | 0.156 |
| Wave 4 | 809.323 | 165 | .000 | 0.88/0.87 | 0.070 [0.065 - 0.075] | 0.000 | 0.146 |
| Wave 5 | 738.666 | 165 | .000 | 0.90/0.88 | 0.068 [0.063 - 0.073] | 0.000 | 0.152 |
| Wave 6 | 716.424 | 165 | .000 | 0.90/0.89 | 0.067 [0.062 - 0.072] | 0.000 | 0.165 |
| Positivity - Adolescent-Intimate partner/ Intimate partner-Adolescent (N7=322, N8= 343, N9=393) | | | | | | | |
|  | *X*^2^ | *df* | Sig. | CFI/TLI | RMSEA [90% C.I.] | RMSEA ≤ .05 | SRMR |
| Wave 7 | 322.222 | 166 | .000 | 0.92/0.91 | 0.054 [0.045 - 0.063] | 0.218 | 0.094 |
| Wave 8 | 308.566 | 166 | .000 | 0.93/0.92 | 0.050 [0.041 - 0.059] | 0.485 | 0.108 |
| Wave 9 | 399.505 | 166 | .000 | 0.91/0.89 | 0.060 [0.052 - 0.067] | 0.016 | 0.112 |
| Engagement - Adolescent-Intimate partner/ Intimate partner-Adolescent (N7=322, N8= 343, N9=393) | | | | | | | |
|  | *X*^2^ | *df* | Sig. | CFI/TLI | RMSEA [90% C.I.] | RMSEA ≤ .05 | SRMR |
| Wave 7 | 380.193 | 165 | .000 | 0.89/0.87 | 0.064 [0.055 - 0.072] | 0.000 | 0.129 |
| Wave 8 | 325.279 | 165 | .000 | 0.92/0.91 | 0.053 [0.045 - 0.062] | 0.259 | 0.116 |
| Wave 9 | 417.430 | 165 | .000 | 0.90/0.88 | 0.062 [0.055 - 0.070] | 0.003 | 0.124 |

*Note.* We used Maximum Likelihood Robust (MLR) as estimator, due to nestedness.

X^2^ = Chi-square, *df* = degrees of freedom, CFI = comparative fit index, TLI = Tucker-Lewis index, RMSEA = root mean square error of approximation, SRMR = standardized root mean square residual.

**Table 5**

*Model fit indices for a second-order factor model with one second-order factor for grouped respondents with nestedness accounted*

| Adolescent-Best friend/ Best friend-Adolescent (N1=911, N2=868, N3=851, N4=834, N5=795, N6=757) | | | | | | | |
| --- | --- | --- | --- | --- | --- | --- | --- |
|  | *X*^2^ | *df* | Sig. | CFI/TLI | RMSEA [90% C.I.] | RMSEA ≤ .05 | SRMR |
| Wave 1 | 440.744 | 164 | 0.000 | 0.92/0.90 | 0.043 [0.038 - 0.048] | 1.991 | 0.070 |
| Wave 4 | 655.745 | 164 | 0.000 | 0.90/0.89 | 0.060 [0.055 - 0.065] | 0.000 | 0.071 |
| Wave 5 | 547.799 | 164 | 0.000 | 0.92/0.91 | 0.054 [0.049 - 0.059] | 0.079 | 0.062 |
| Wave 6 | 420.906 | 164 | 0.000 | 0.95/0.94 | 0.045 [0.040 - 0.051] | 0.915 | 0.054 |
| Adolescent-Mother/ Adolescent-Father/ Sibling-Mother (N1=1378, N2=1316, N3=1272, N4=1237, N5=1187, N6=1187) | | | | | | | |
|  | *X*^2^ | *df* | Sig. | CFI/TLI | RMSEA [90% C.I.] | RMSEA ≤ .05 | SRMR |
| Wave 1 | 646.465 | 164 | 0.000 | 0.91/0.89 | 0.046 [0.042 - 0.050] | 0.951 | 0.071 |
| Wave 4 | 765.677 | 164 | 0.000 | 0.92/0.91 | 0.054 [0.051 - 0.058] | 0.029 | 0.073 |
| Wave 5 | 782.013 | 164 | 0.000 | 0.92/0.90 | 0.056 [0.052 - 0.060] | 0.004 | 0.075 |
| Wave 6 | 420.906 | 164 | 0.000 | 0.95/0.94 | 0.045 [0.040 - 0.051] | 0.915 | 0.054 |
| Mother-Adolescent/ Father-Adolescent (N1=940, N2=885, N3=868, N4=838, N5=794, N6=792) | | | | | | | |
|  | *X*^2^ | *df* | Sig. | CFI/TLI | RMSEA [90% C.I.] | RMSEA ≤ .05 | SRMR |
| Wave 1 | 543.091 | 164 | 0.000 | 0.91/0.90 | 0.050 [0.045 - 0.054] | 0.550 | 0.060 |
| Wave 2 | 551.167 | 164 | 0.000 | 0.91/0.90 | 0.052 [0.047 - 0.056] | 0.277 | 0.059 |
| Wave 3 | 558.259 | 164 | 0.000 | 0.91/0.90 | 0.053 [0.048 - 0.057] | 0.179 | 0.060 |
| Wave 4 | 573.898 | 164 | 0.000 | 0.91/0.89 | 0.055 [0.050 - 0.060] | 0.058 | 0.063 |
| Wave 5 | 451.180 | 164 | 0.000 | 0.94/0.93 | 0.047 [0.042 - 0.052] | 0.829 | 0.057 |
| Wave 6 | 585.560 | 164 | 0.000 | 0.91/0.90 | 0.057 [0.052 - 0.062] | 0.011 | 0.059 |
| Mother-Father/ Father-Mother (N1=893, N2=848, N3=827, N4=801, N5=744, N6=740) | | | | | | | |
|  | *X*^2^ | *df* | Sig. | CFI/TLI | RMSEA [90% C.I.] | RMSEA ≤ .05 | SRMR |
| Wave 1 | 704.938 | 164 | 0.000 | 0.91/0.90 | 0.061 [0.056 - 0.065] | 0.000 | 0.075 |
| Wave 2 | 749.920 | 164 | 0.000 | 0.90/0.88 | 0.065 [0.060 - 0.070] | 0.000 | 0.076 |
| Wave 3 | 704.991 | 164 | 0.000 | 0.91/0.90 | 0.063 [0.058 - 0.068] | 0.000 | 0.070 |
| Wave 4 | 639.331 | 164 | 0.000 | 0.91/0.90 | 0.060 [0.055 - 0.065] | 0.000 | 0.067 |
| Wave 5 | 579.024 | 164 | 0.000 | 0.93/0.92 | 0.058 [0.053 - 0.064] | 0.004 | 0.062 |
| Wave 6 | 527.116 | 164 | 0.000 | 0.94/0.93 | 0.055 [0.050 - 0.060] | 0.068 | 0.059 |
| Adolescent-Intimate partner/ Intimate partner-Adolescent (N7=322, N8= 343, N9=393) | | | | | | | |
|  | *X*^2^ | *df* | Sig. | CFI/TLI | RMSEA [90% C.I.] | RMSEA ≤ .05 | SRMR |
| Wave 7 | 317.121 | 164 | 0.000 | 0.92/0.91 | 0.054 [0.045 - 0.063] | 0.231 | 0.083 |
| Wave 8 | 280.411 | 164 | 0.000 | 0.94/0.94 | 0.045 [0.036 - 0.054] | 0.789 | 0.076 |
| Wave 9 | 361.092 | 164 | 0.000 | 0.92/0.91 | 0.055 [0.048 - 0.063] | 0.126 | 0.073 |

*Note.* We used Maximum Likelihood Robust (MLR) as estimator, due to nestedness.

X^2^ = Chi-square, *df* = degrees of freedom, CFI = comparative fit index, TLI = Tucker-Lewis index, RMSEA = root mean square error of approximation, SRMR = standardized root mean square residual.

**Table 6**

*Model fit indices for bifactor models with reference domain (S-1)^1^ for grouped respondents with nestedness accounted*

| Adolescent-Best friend/ Best friend-Adolescent (N1=911, N2=868, N3=851, N4=834, N5=795, N6=757) | | | | | | | |
| --- | --- | --- | --- | --- | --- | --- | --- |
|  | *X*^2^ | *df* | Sig. | CFI/TLI | RMSEA [90% C.I.] | RMSEA ≤ .05 | SRMR |
| Wave 1 | 326.340 | 149 | 0.000 | 0.95/0.93 | 0.036 [0.031 - 0.041] | 1.000 | 0.047 |
| Wave 4 | 499.989 | 149 | 0.000 | 0.93/0.91 | 0.053 [0.048 - 0.058] | 0.152 | 0.045 |
| Wave 5 | 467.386 | 149 | 0.000 | 0.93/0.92 | 0.052 [0.047 - 0.057] | 0.276 | 0.043 |
| **Wave 6** | **341.401** | **149** | **0.000** | **0.96/0.95** | **0.041 [0.036 - 0.047]** | **0.994** | **0.037** |
| Adolescent-Mother/ Adolescent-Father/ Sibling-Mother (N1=1378, N2=1316, N3=1272, N4=1237, N5=1187, N6=1187) | | | | | | | |
|  | *X*^2^ | *df* | Sig. | CFI/TLI | RMSEA [90% C.I.] | RMSEA ≤ .05 | SRMR |
| Wave 1 | 516.323 | 149 | 0.000 | 0.93/0.91 | 0.042 [0.038 - 0.046] | 0.999 | 0.051 |
| Wave 4 | 615.211 | 149 | 0.000 | 0.93/0.92 | 0.050 [0.046 - 0.054] | 0.445 | 0.049 |
| Wave 5 | 628.649 | 149 | 0.000 | 0.93/0.92 | 0.052 [0.048 - 0.056] | 0.203 | 0.047 |
| Wave 6 | 579.013 | 149 | 0.000 | 0.95/0.93 | 0.049 [0.045 - 0.054] | 0.598 | 0.048 |
| Mother-Adolescent/ Father-Adolescent (N1=940, N2=885, N3=868, N4=838, N5=794, N6=792) | | | | | | | |
|  | *X*^2^ | *df* | Sig. | CFI/TLI | RMSEA [90% C.I.] | RMSEA ≤ .05 | SRMR |
| Wave 1 | 394.154 | 149 | 0.000 | 0.94/0.93 | 0.042 [0.037 - 0.047] | 0.996 | 0.038 |
| Wave 2 | 434.538 | 149 | 0.000 | 0.94/0.92 | 0.047 [0.041 - 0.052] | 0.865 | 0.040 |
| Wave 3 | 433.388 | 149 | 0.000 | 0.94/0.92 | 0.047 [0.042 - 0.052] | 0.835 | 0.042 |
| Wave 4 | 446.254 | 149 | 0.000 | 0.93/0.91 | 0.049 [0.044 - 0.054] | 0.640 | 0.042 |
| **Wave 5** | **335.918** | **149** | **0.000** | **0.96/0.95** | **0.040 [0.034 - 0.045]** | **0.999** | **0.035** |
| Wave 6 | 461.532 | 149 | 0.000 | 0.94/0.92 | 0.051 [0.046 - 0.057] | 0.318 | 0.039 |
| Mother-Father/ Father-Mother (N1=893, N2=848, N3=827, N4=801, N5=744, N6=740) | | | | | | | |
|  | *X*^2^ | *df* | Sig. | CFI/TLI | RMSEA [90% C.I.] | RMSEA ≤ .05 | SRMR |
| Wave 1 | 513.631 | 149 | 0.000 | 0.94/0.93 | 0.052 [0.047 - 0.057] | 0.211 | 0.044 |
| Wave 2 | 595.994 | 149 | 0.000 | 0.92/0.90 | 0.059 [0.055 - 0.065] | 0.001 | 0.050 |
| Wave 3 | 567.786 | 149 | 0.000 | 0.93/0.91 | 0.058 [0.053 - 0.063] | 0.004 | 0.046 |
| Wave 4 | 496.696 | 149 | 0.000 | 0.94/0.92 | 0.054 [0.049 - 0.059] | 0.103 | 0.043 |
| Wave 5 | 457.073 | 149 | 0.000 | 0.95/0.93 | 0.053 [0.047 - 0.058] | 0.202 | 0.038 |
| Wave 6 | 425.120 | 149 | 0.000 | 0.95/0.94 | 0.050 [0.044 - 0.056] | 0.485 | 0.038 |
| Adolescent-Intimate partner/ Intimate partner-Adolescent (N7=322, N8= 343, N9=393) | | | | | | | |
|  | *X*^2^ | *df* | Sig. | CFI/TLI | RMSEA [90% C.I.] | RMSEA ≤ .05 | SRMR |
| Wave 7 | 246.280 | 149 | 0.000 | 0.95/0.94 | 0.045 [0.035 - 0.055] | 0.789 | 0.051 |
| Wave 8 | 226.138 | 149 | 0.000 | 0.96/0.95 | 0.039 [0.028 - 0.039] | 0.968 | 0.047 |
| Wave 9 | 307.004 | 149 | 0.000 | 0.94/0.92 | 0.052 [0.044 - 0.060] | 0.339 | 0.049 |

Note. We used Maximum Likelihood Robust (MLR) as estimator, due to nestedness.

^1^Model S-1 refers to a bifactor model with withdrawal as reference dimension for the general factor, and partial correlations between the specific factors.

X^2^ = Chi-square, *df* = degrees of freedom, CFI = comparative fit index, TLI = Tucker-Lewis index, RMSEA = root mean square error of approximation, SRMR = standardized root mean square residual. Bolded models represent bifactor models with coefficients detailed in Figure 5 and Figure 6 respectively.

**Table 7**

*Standardized factor loadings for the general and specific factors in the S-1 with withdrawal as reference domain bifactor models per type of respondent*

|  | *Estimates for adolescents* | |  | *Estimates for young adults* | |  | *Estimates for adults* | |
| --- | --- | --- | --- | --- | --- | --- | --- | --- |
| *Item* | *β* | *S.E.* |  | *β* | *S.E.* |  | *Β* | *S.E.* |
| *General factor* |  |  |  |  |  |  |  |  |
| 1. Personally attack him/her | .235 - .340 | .034 - .044 |  | .260 - .317 | .051 - .066 |  | .254 - .348 | .037 - .040 |
| 2. Focusing on the problem at hand | -.138 - .022 | .037 - .052 |  | -.128 - -.274 | .053 - .073 |  | -.278 - -.375 | .035 - .043 |
| 3. Remaining silent for long periods of time | .330 - .628 | .025 - .051 |  | .509 - .626 | .039 - .059 |  | .422 - .681 | .024 - .037 |
| 4. *Not being willing to stick up for myself | .164 - .425 | .034 - .042 |  | .255 - .380 | .053 - .064 |  | .218 - .520 | .037 - .041 |
| 5. Exploding and getting out of control | .356 - .505 | .032 - .037 |  | .304 - .366 | .059 - .063 |  | .267 - .376 | .035 - .041 |
| 6. Sitting down and discussing differences constructively | -.092 - -.288 | .034 - .039 |  | -.297 - -.379 | .051 - .071 |  | -.304 - -.528 | .032 - .038 |
| 7. Reaching a limit, “shutting down,” and refusing to talk any further | .501 - .717 | .022 - .037 |  | .632 - .776 | .033 - .048 |  | .678 - .799 | .018 - .035 |
| 8. Being too compliant | .153 - .451 | .034 - .047 |  | .107 - .233 | .051 - .065 |  | .221 - .409 | .036 - .043 |
| 9. Getting carried away and saying things that aren’t meant | .432 - .621 | .031 - .037 |  | .366 - .425 | .054 - .058 |  | .365 - .438 | .034 - .039 |
| 10. Finding alternatives that are acceptable to each of us | -.023 - -.312 | .035 - .039 |  | -.266 - -.317 | .056 - .077 |  | -.281 - -.449 | .033 - .043 |
| 11. Tuning the other person out | .699 - .803 | .021 - .032 |  | .624 - .757 | .039 - .047 |  | .677 - .795 | .020 - .029 |
| 12. *Not defending my position | .202 - .460 | .032 - .043 |  | .236 - .264 | .058 - .070 |  | .344 - .510 | .036 - .040 |
| 13. Throwing insults and digs | .387 - .516 | .031 - .037 |  | .302 - .546 | .048 - .056 |  | .401 - .465 | .034 - .041 |
| 14. Negotiating and compromising | -.041 - - 261 | .034 - .038 |  | -.193 - -.291 | .054 - .074 |  | -.142 - -.421 | .036 - .043 |
| 15. Withdrawing, acting distant and not interested | .693 - .789 | .018 - .032 |  | .695 - .744 | .032 - .041 |  | .669 - .814 | .019 - .027 |
| 16. Giving in with little attempt to present my side of the issue | .238 - .440 | .033 - .049 |  | .269 - .282 | .055 - .062 |  | .305 - .449 | .034 - .043 |
| 17. Getting so angry that I do not know what I am doing anymore | .345 - .506 | .030 - .043 |  | .361 - .407 | .053 - .063 |  | .251 - .381 | .034 - .056 |
| 18. Searching for a solution that is good for both of us | -.059 - -.339 | .035 - .045 |  | -.252 - -.341 | .063 - .079 |  | -.284 - -.429 | .034 - .045 |
| 19. *Not responding to him/her anymore | .740 - .847 | .016 - .036 |  | .814 - .840 | .025 - .038 |  | .720 - 815 | .016 - .023 |
| 20. Let him/her have his/her own way | .138 - .406 | .033 - .049 |  | .100 - .164 | .052 - .067 |  | .179 - .318 | .036 - .043 |
| *Conflict engagement* |  |  |  |  |  |  |  |  |
| 1. Personally attack him/her | .361 - .594 | .042 - .080 |  | .429 - .545 | .055 - .076 |  | .364 - .670 | .029 - .046 |
| 5. Exploding and getting out of control | .481 - .607 | .037 - .080 |  | .583 - .646 | .044 - .067 |  | .556 - .686 | .032 - .048 |
| 9. Getting carried away and saying things that aren’t meant | .324 - .677 | .035 - .081 |  | .622 - .707 | .046 - .066 |  | .602 - .710 | .034 - .042 |
| 13. Throwing insults and digs | .409 - .599 | .037 - .069 |  | .428 - .633 | .049 - .063 |  | .350 - .640 | .033 - .044 |
| 17. Getting so angry that I do not know what I am doing anymore | .379 - .611 | .043 - .088 |  | .360 - .496 | .062 - .083 |  | .445 - .531 | .035 - .048 |
| *Positive problem solving* |  |  |  |  |  |  |  |  |
| 2. Focusing on the problem at hand | .385 - .655 | .030 - .044 |  | .446 - .508 | .061 - .080 |  | .326 - .521 | .036 - .047 |
| 6. Sitting down and discussing differences constructively | .592 - .737 | .021 - .030 |  | .560 - .648 | .049 - .070 |  | .459 - .564 | .031 - .040 |
| 10. Finding alternatives that are acceptable to each of us | .780 - .862 | .017 - .024 |  | .720 - .829 | .035 - .050 |  | .678 - .767 | .025 - .040 |
| 14. Negotiating and compromising | .734 - .836 | .017 - .025 |  | .690 - .740 | .038 - .065 |  | .578 - .710 | .029 - .042 |
| 18. Searching for a solution that is good for both of us | .820 - .877 | .015 - .029 |  | .773 - .816 | .034 - .044 |  | .755 - .846 | .022 - .034 |
| *Compliance* |  |  |  |  |  |  |  |  |
| 4. *Not being willing to stick up for myself | .312 - .542 | .034 - .055 |  | .496 - .630 | .055 - .074 |  | .283 - .605 | .042 - .085 |
| 8. Being too compliant | .543 - .676 | .034 - .063 |  | .604 - .704 | .047 - .078 |  | .400 - .529 | .042 - .060 |
| 12. *Not defending my position | .352 - .529 | .037 - .065 |  | .529 - .609 | .051 - .071 |  | .259 - .627 | .041 - .081 |
| 16. Giving in with little attempt to present my side of the issue | .430 - .604 | .033 - .059 |  | .559 - .609 | .052 - .063 |  | .533 - .686 | .042 - .085 |
| 20. Let him/her have his/her own way | .466 - .548 | .035 - .059 |  | .490 -.599 | .057 - .082 |  | .368 - .560 | .043 - .066 |

*Note.* *items coded on the reverse.

More than 99% of the factor loadings on the general factor in adults were significant. More than 93% of the factor loadings on the general factor in adolescents were significant. All the non-significant factor loadings on the general factor regarded items belonging to the positive problem-solving dimension. All factor loadings for the specific factors were significant. **In youths, about 50% of the factor loadings on the general factor were ≥.40, and 64% of them were ≥.30.** **In adults, about 47% of the factor loadings were ≥.40, and about 80% of the factor loadings were ≥.30.**

**Table 8**

*Ranges of standardized regression coefficients for the predictors and outcomes of conflict management dimensions in adults (mother-father, father-mother, mother-adolescent, father-adolescent)*

|  | | | | | | | | |
| --- | --- | --- | --- | --- | --- | --- | --- | --- |
| **Predictors (factors) of conflict management dimensions** | | | | | | | | |
| *Conflict intensity* | | | |  |  | | | |
|  | Β | S.E. | *p* |  |  |  |  |  |
| General factor | .210 - .321 | .037 - .042 | .000 |  |  |  |  |  |
| Conflict engagement | .100 - .304 | .042 - .051 | .000 - .051 |  |  |  |  |  |
| Positive problem solving | .001 - .109 | .039 - .049 | .011 - .978 |  |  |  |  |  |
| Compliance | -.010 - .093 | .051 - .065 | .067 - .861 |  |  |  |  |  |
| *Support (NRI)* | | | |  | *Negativity (NRI)* | | | |
|  | Β | S.E. | *p* |  |  | β | S.E. | *P* |
| General factor | -.163 - -.283 | .036 - .038 | .000 |  | General factor | .205 - .331 | .042 - .063 | .000 |
| Conflict engagement | -.003 - .125 | .043 - .050 | .008 - .948 |  | Conflict engagement | .276 - .398 | .046 - .076 | .000 |
| Positive problem solving | .189 - .345 | .039 - .049 | .000 |  | Positive problem solving | -.040 - -.161 | .041 - .056 | .002 - .265 |
| Compliance | -.050 - -.133 | .042 - .054 | .001 - .200 |  | Compliance | -.111 - -.295 | .048 - .060 | .000 - .047 |
| *Power (NRI)* | | | |  |  | | | |
|  | Β | S.E. | *p* |  |  |  |  |  |
| General factor | .030 - .182 | .035 - .042 | .001 - .479 |  |  |  |  |  |
| Conflict engagement | -.002 - -.195 | .037 - .050 | .000 - .962 |  |  |  |  |  |
| Positive problem solving | .001 - .109 | .036 - .065 | .003 - .987 |  |  |  |  |  |
| Compliance | .421 - .549 | .038 - .065 | .000 |  |  |  |  |  |
| **Outcomes of conflict management dimensions** | | | | | | | | |
| *Internalizing problems* | | | |  | *Externalizing problems* | | | |
|  | Β | S.E. | *p* |  |  | β | S.E. | *P* |
| General factor | .163 - .293 | .036 - .043 | .000 |  | General factor | .181 - .323 | .035 - .046 | .000 |
| Conflict engagement | .123 - .245 | .043 - .063 | .000 - .052 |  | Conflict engagement | .225 - .367 | .041 - .055 | .000 |
| Positive problem solving | -.002 - -.027 | .043 - .049 | .571 - .893 |  | Positive problem solving | -.015 - -.079 | .037 - .047 | .068 - .690 |
| Compliance | .138 - .250 | .048 - .079 | .000 - .041 |  | Compliance | .032 - .138 | .045 - .070 | .005 - .562 |
| *Prosocial behaviours* | | | |  |  |  |  |  |
|  | Β | S.E. | *p* |  |  |  |  |  |
| General factor | -.124 - -.299 | .034 - .041 | .000 |  |  |  |  |  |
| Conflict engagement | -.003 - .089 | .038 - .051 | .060 - .946 |  |  |  |  |  |
| Positive problem solving | .108 - .252 | .039 - .052 | .000 - .009 |  |  |  |  |  |
| Compliance | -.024 - -.182 | .042 - .050 | .000 - .614 |  |  |  |  |  |

*Note*. Β = standardized regression coefficient, S.E. = Standard Error, *p* = significance level.

**Table 9**

*Ranges of standardized regression coefficients for the predictors and outcomes of conflict management dimensions in adolescents (adolescent-best friend, best friend-adolescent, adolescent-mother, adolescent-father, sibling-mother)*

|  | | | | | | | | |
| --- | --- | --- | --- | --- | --- | --- | --- | --- |
| **Predictors (factors) of conflict management dimensions** | | | | | | | | |
| *Conflict intensity* | | | |  |  | | | |
|  | Β | S.E. | *p* |  |  |  |  |  |
| General factor | .178 - .304 | .033 - .048 | .000 |  |  |  |  |  |
| Conflict engagement | .167 - .288 | .045 - .068 | .000 - .009 |  |  |  |  |  |
| Positive problem solving | .005 - .069 | .035 - .039 | .073 - .901 |  |  |  |  |  |
| Compliance | -.083 - .135 | .046 - .057 | .004 - .833 |  |  |  |  |  |
| *Support (NRI)* | | | |  | *Negativity (NRI)* | | | |
|  | Β | S.E. | *p* |  |  | β | S.E. | *P* |
| General factor | -.057 - -.203 | .036 - .044 | .000 - .189 |  | General factor | .153 - .310 | .038 - .047 | .000 - .001 |
| Conflict engagement | -.021 - -.086 | .045 - .050 | .087 - .651 |  | Conflict engagement | .174 - .449 | .045 - .072 | .000 - .012 |
| Positive problem solving | .117 - .315 | .034 - .043 | .000 - .007 |  | Positive problem solving | -.044 - -.122 | .037 - .041 | .003 - .262 |
| Compliance | .001 - -.063 | .044 - .050 | .171 - .983 |  | Compliance | -.109 - -.191 | .043 - .052 | .000 - .038 |
| *Power (NRI)* | | | |  |  | | | |
|  | Β | S.E. | *p* |  |  |  |  |  |
| General factor | .066 - .118 | .033 - .044 | .002 - .109 |  |  |  |  |  |
| Conflict engagement | -.009 - -.089 | .045 - .051 | .062 - .853 |  |  |  |  |  |
| Positive problem solving | -.020 - .044 | .036 - .041 | .280 - .862 |  |  |  |  |  |
| Compliance | .147 - .268 | .043 - .053 | .000 - .001 |  |  |  |  |  |
| **Outcomes of conflict management dimensions** | | | | | | | | |
| *Internalizing problems* | | | |  | *Externalizing problems* | | | |
|  | Β | S.E. | *p* |  |  | β | S.E. | *P* |
| General factor | .201 - .378 | .037 - .046 | .000 |  | General factor | .195 - .248 | .036 - .048 | .000 |
| Conflict engagement | .027 - .268 | .045 - .080 | .000 - .623 |  | Conflict engagement | .203 - .306 | .046 - .072 | .000 - .002 |
| Positive problem solving | -.066 - .070 | .037 - .048 | .087 - .647 |  | Positive problem solving | -.007 - -.127 | .040 - .051 | .000 - .904 |
| Compliance | .049 - .231 | .049 - .072 | .000 - .455 |  | Compliance | -.079 - .081 | .046 - .064 | .089 - .943 |
|  |  |  |  |  |  |  |  |  |
| *Prosocial behaviours* | | | |  |  |  |  |  |
|  | Β | S.E. | *p* |  |  |  |  |  |
| General factor | -.058 - -.106 | .035 - .043 | .004 - .138 |  |  |  |  |  |
| Conflict engagement | -.015 - -.155 | .037 - .048 | .001 - .752 |  |  |  |  |  |
| Positive problem solving | .098 - .165 | .040 - .052 | .000 - .028 |  |  |  |  |  |
| Compliance | -.018 - -.138 | .046 - .059 | .012 - .717 |  |  |  |  |  |

*Note*. Β = standardized regression coefficient, S.E. = Standard Error, *p* = significance level.

**Table 10**

*Ranges of standardized regression coefficients for the predictors and outcomes of conflict management dimensions in young adults (target young adult-romantic partner, romantic partner-target young adult)*

|  | | | | | | | | |
| --- | --- | --- | --- | --- | --- | --- | --- | --- |
| **Predictors (factors) of conflict management dimensions** | | | | | | | | |
| *Conflict intensity* | | | |  |  | | | |
|  | Β | S.E. | *p* |  |  |  |  |  |
| General factor | .117 - .206 | .078 - .091 | .024 - .135 |  |  |  |  |  |
| Conflict engagement | .260 - .307 | .090 - .098 | .001 - .008 |  |  |  |  |  |
| Positive problem solving | .019 - .036 | .082 - .089 | .658 - .831 |  |  |  |  |  |
| Compliance | .011 - -.049 | .086 - .087 | .570 - .896 |  |  |  |  |  |
| *Support (NRI)* | | | |  | *Negativity (NRI)* | | | |
|  | Β | S.E. | *P* |  |  | β | S.E. | *p* |
| General factor | -.085 - -.142 | .064 - .072 | .049 - .186 |  | General factor | .042 - .153 | .091 - .098 | .091 - .665 |
| Conflict engagement | .004 - .162 | .066 - .095 | .014 - .964 |  | Conflict engagement | .320 - .397 | .092 - .095 | .000 - .001 |
| Positive problem solving | -.020 - .031 | .066 | .638 - .760 |  | Positive problem solving | -.088 - -.137 | .078 - .083 | .101 - .259 |
| Compliance | -.177 - -.247 | .072 - .087 | .005 - .014 |  | Compliance | -.093 - -.128 | .074 - .089 | .148 - .205 |
| *Power (NRI)* | | | |  |  | | | |
|  | Β | S.E. | *p* |  |  |  |  |  |
| General factor | .083 - .194 | .071 - .087 | .006 - .341 |  |  |  |  |  |
| Conflict engagement | -.096 - -.127 | .080 - .082 | .111 - .242 |  |  |  |  |  |
| Positive problem solving | .003 - .112 | .071 - .073 | .121 - .967 |  |  |  |  |  |
| Compliance | .273 - .296 | .077 - .093 | .000 - .003 |  |  |  |  |  |
| **Outcomes of conflict management dimensions** | | | | | | | | |
| *Internalizing problems* | | | |  | *Externalizing problems* | | | |
|  | Β | S.E. | *p* |  |  | β | S.E. | *P* |
| General factor | .108 - .299 | .067 - .078 | .000 - .165 |  | General factor | .152 - .305 | .068 - .078 | .000 - .035 |
| Conflict engagement | .167 - .277 | .089 - .094 | .003 - .060 |  | Conflict engagement | .178 - .190 | .081 - .096 | .028 - .049 |
| Positive problem solving | -.089 - -.102 | .074 - .076 | .175 - .215 |  | Positive problem solving | -.059 - .063 | .064 - .080 | .326 - .459 |
| Compliance | .157 - .293 | .084 - .097 | .000 - .107 |  | Compliance | .059 - .124 | .069 - .100 | .071 - .556 |
| *Prosocial behaviours* | | | |  |  |  |  |  |
|  | Β | S.E. | *p* |  |  |  |  |  |
| General factor | -.073 - -.144 | .058 - .063 | .022 - .209 |  |  |  |  |  |
| Conflict engagement | -.062 - -.071 | .065 - .116 | .346 - .540 |  |  |  |  |  |
| Positive problem solving | .117 - .126 | .067 - .079 | .060 - .142 |  |  |  |  |  |
| Compliance | -.054 - -.232 | .064 - .082 | .005 - .399 |  |  |  |  |  |

*Note*. Β = standardized regression coefficient, S.E. = Standard Error, *p* = significance level.
